# Fibroblast Growth Factor Receptor 2 Isoforms Detected via Novel RNA ISH as Predictive Biomarkers for Progestin Therapy in Atypical Hyperplasia and Low-Grade Endometrial Cancer

**DOI:** 10.3390/cancers13071703

**Published:** 2021-04-03

**Authors:** Asmerom T. Sengal, Deborah Smith, Rebecca Rogers, Cameron E. Snell, Elizabeth D. Williams, Pamela M. Pollock

**Affiliations:** 1 School of Biomedical Sciences, Faculty of Health, Queensland University of Technology, Translational Research Institute, Princess Alexandra (PA) Hospital Campus, 37 Kent St., Woolloongabba, Brisbane, Queensland 4102, Australia; asmerom.sengal@qut.edu.au (A.T.S.); ed.williams@qut.edu.au (E.D.W.); 2 Mater Pathology, Mater Research and University of Queensland, Mater Hospital, Raymond Terrace, South Brisbane, Queensland 4101, Australia; Deborah.Smith@mater.org.au (D.S.); Cameron.Snell@mater.org.au (C.E.S.); 3 Mater Pathology, Mater Research, Mater Hospital, Raymond Terrace, South Brisbane, Queensland 4101, Australia; rebecca.rogers@mater.uq.edu.au

**Keywords:** atypical hyperplasia, endometrioid endometrial cancer, biomarkers, FGFR2b, FGFR2c, LNG-IUD/Mirena, RNA ISH

## Abstract

**Simple Summary:**

Women diagnosed with low-grade endometrioid cancer (EEC) and its precursor lesion, atypical hyperplasia (AH) are frequently treated with hormonal therapy including levonorgestrel releasing intrauterine device (LNG-IUD) as an alternative to surgery. Biomarkers that inform which group of patients are more likely to respond to LNG-IUD are not available. The aim of this study was to document the response rate to LNG-IUD therapy in women with AH and EEC and identify potential biomarkers to guide treatment response. The overall response rate (ORR) for the whole cohort was 30/69 (~44%) with a higher ORR seen in AH (64%) compared to EEC (23%). Fibroblast Growth Factor Receptor (FGFR2) isoforms were detected using RNA in situ hybridization. The FGFR2c isoform was expressed in 16.7% of the samples, with those expressing FGFR2c 5-times more likely to have treatment failure. FGFR2 isoform expression could be used to guide treatment decisions following confirmation of this finding in an independent study.

**Abstract:**

Women with atypical hyperplasia (AH) or well-differentiated early-stage endometrioid endometrial carcinoma (EEC) who wish to retain fertility and/or with comorbidities precluding surgery, are treated with progestin. Clinically approved predictive biomarkers for progestin therapy remain an unmet need. The objectives of this study were to document the overall response rate (ORR) of levonorgestrel intrauterine device (LNG-IUD) treatment, and determine the association of FGFR2b and FGFR2c expression with treatment outcome. BaseScope RNA ISH assay was utilized to detect expression of FGFR2b and FGFR2c mRNA in the diagnostic biopsies of 89 women (40 AH and 49 EEC) treated with LNG-IUD. Detailed clinical follow-up was available for 69 women which revealed an overall response rate (ORR) of 44% (30/69) with a higher ORR seen in AH (64%) compared to EEC (23%). The recurrence rate in women who initially responded to LNG-IUD was 10/30 (33.3%). RNA ISH was successful in 72 patients and showed FGFR2c expression in 12/72 (16.7%) samples. In the 59 women with detailed clinical follow-up and RNA-ISH data, women with tumours expressing FGFR2c were 5-times more likely to have treatment failure in both univariable (HR 5.08, *p* < 0.0001) and multivariable (HR 4.5, *p* < 0.002) Cox regression analyses. In conclusion, FGFR2c expression appears to be strongly associated with progestin treatment failure, albeit the ORR is lower in this cohort than previously reported. Future work to validate these findings in an independent multi-institutional cohort is needed.

## 1. Introduction

Endometrial carcinoma (EC) is the most frequent gynaecologic malignancy in developed countries and the annual incidence and mortality rates are increasing [1]. Endometrioid EC (EEC) is the most common histologic type and is commonly associated with obesity [2]. Endometrial hyperplasia is considered the precursor lesion of EEC and usually develops after exogenous or endogenous unopposed excess estrogen exposure. Since 2014 the WHO classification of endometrial hyperplasia has been divided into benign hyperplasia and atypical hyperplasia (AH) that have 1–3% and 30–40% risk of progression to EEC, respectively if left untreated [3,4,5].

Women with AH and well-differentiated early-stage EEC who prefer to preserve fertility, or with comorbidities that preclude surgery, are treated with progestin as an alternative to the standard of care total hysterectomy. Progestin regimens frequently used include oral medroxyprogesterone acetate (MPA), megestrol acetate (MA) or a levonorgestrel releasing intrauterine device (LNG-IUD)/Mirena^®^ [6]. LNG-IUD has been used increasingly and is considered preferable as compared to oral progestin due to better tolerance (fewer side effects) and increased compliance [7,8]. The reported response rates for LNG-IUD therapy are ~80% for AH and ~60% for EEC [8,9], however reported relapse rates are high (22–41%) [10,11,12].

Clinically validated predictive biomarkers to identify those patients unlikely to respond to progestin or who are most likely to relapse after initial response, are not available and this is unmet need in clinical practice. Several studies have investigated the role of progesterone receptor (PR) expression for predicting progestin treatment response in AH and well-differentiated EEC, but still, there is no consensus in the reported studies [13].

**Figure 1:**
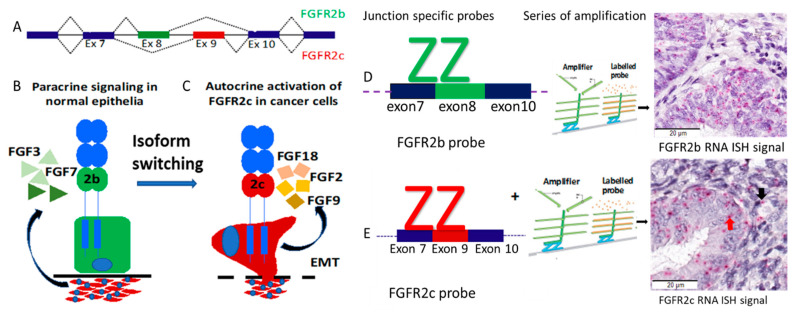
FGFR2 isoform switching, paracrine and autocrine signalling, and RNA ISH probes design. (**A**) Mechanism of alternative splicing. FGFR2 has two alternative spliced isoforms, FGFR2b and FGFR2c and these differ only in the second half of the third immunoglobulin-like loop (Ig-III) in the extracellular domain. Inclusion of exon 8 and exclusion of exon 9 gives rise to the FGFR2b “2b” isoform and inclusion of exon 9 and exclusion of exon 8 gives rise to the FGFR2c “2c” isoform. (**B**) The FGFR2b isoform is normally expressed in epithelial cells and stimulated by FGF ligands (for example, FGF3, FGF7) expressed by underlying stromal cells. (**C**) Carcinoma cells undergo isoform switching during progression resulting in autocrine receptor activation. (**D**, **E**) Principles of probe design and representative micrography of RNA ISH showing (**D**) FGFR2b isoform (**E**) FGFR2c isoform signals. FGF, Fibroblast Growth Factor; FGFR2, Fibroblast Growth Factor Receptor.

Dysregulation of Fibroblast Growth Factor (FGF)/FGFR2 signalling has been shown to contribute to the progression of EEC [14,15,16,17]. *FGFR2* is a prototype gene that undergoes alternative splicing and generates two major isoforms which show mutually exclusive tissue-specific splicing and expression. FGFR2b includes exon 8 and is normally expressed in epithelial derived cells whereas FGFR2c includes exon 9 and is expressed in mesenchymal derived cells (Figure 1A). Epithelial-mesenchymal FGF signalling normally occurs in a reciprocal fashion where the ligands specific to each isoform are expressed by the other tissue type to drive paracrine receptor signalling. Specifically, normal epithelial cells express FGFR2b which can be stimulated by FGF3, 7, 10 and 22 that are expressed by the underlying mesenchymal cells [18] (Figure 1B), and FGFR2c expressed by the stroma binds with FGF2, 4, 8, 9, 17, 18 and 20 which can be expressed by epithelial cells. In carcinoma cells FGFR2 undergoes isoform switching from FGFR2b to FGFR2c during tumorigenesis which results in constitutive receptor activation via autocrine signalling (Figure 1C). This results in functional and phenotypic changes including increased motility, resistance to apoptosis, stemness, and epithelial to mesenchymal transition (EMT) [19,20,21]. In contrast, FGFR2b is associated with well differentiated tumours, good outcome and has been shown to have a tumour suppressive role in some cancers [22,23].

Recently, we found FGFR2c expression is associated with aggressive clinicopathologic markers (high grade, deep myometrial invasion and lymphovascular space invasion) and shorter progression-free survival (PFS) and disease-specific survival (DSS) in EEC [24]. We hypothesised that FGFR2 isoform switching occurs in a subset of AH and early-stage EEC and contributes to progestin treatment failure or resistance. The objectives of this study were i) to determine the response rates of LNG-IUD treatment in a cohort of patients with AH and EEC and ii) to investigate the association of FGFR2b and FGFR2c expression with outcome in women with AH and well-differentiated early-stage EEC who had been treated with LNG-IUD at the Mater Hospital.

## 2. Results

### 2.1. Patient Cohort Characteristics and Treatment Outcomes

**Figure 2:**
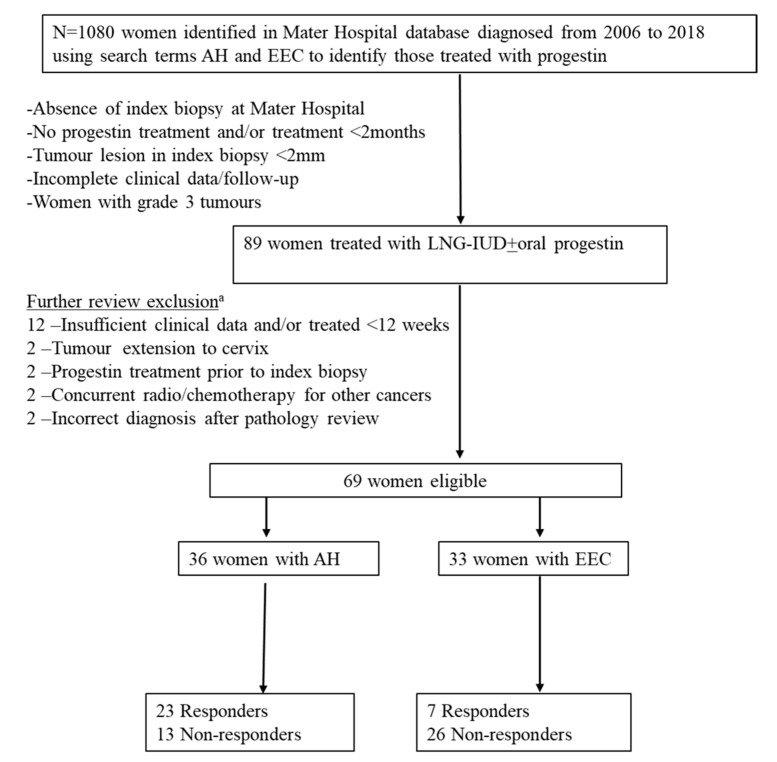
REMARK flow chart of the cohort. ^a^: 20 women were excluded on further review of the 89 cases. AH, atypical hyperplasia; EEC, endometrioid endometrial carcinoma.

Of the 89 women treated with LNG-IUD, 69 (77.5%) had complete clinical and follow-up data, resulting in 36 AH, and 33 EECs including 4 women with grade 2 EEC. A total of 20 women with AH (n = 4), grade 1 EEC (n = 13) and grade 2 EEC (n = 3) were dropped from subsequent outcome analyses as they did not fulfill the criteria set for the study (Figure 2).

The indications for conservative progestin treatment were desire to preserve fertility (n = 10), comorbidity that preclude surgery including morbid obesity increasing surgical risk (n = 40), patient preferences (n = 6) and symptom control until hysterectomy was feasible ± comorbidity (n = 26). The ages ranged from 26 to 86 years with a mean (±SEM) of 54.4 ± 1.56 and a median age of 56 years. There were 20 women under 50 years. The mean (±SEM) BMI was 47.7 (±1.34), 95% CI, 35.7–55.5 and median BMI was 48.4 Kg/m^2^. The median follow-up was 524 days with interquartile range (IQR) of 386–1286 days. All women were treated with LNG-IUD with 11 (14%) and 20 (29%) receiving additional oral progestin or Metformin treatment, respectively.

The association between clinicopathologic characteristics and outcomes in LNG-IUD responders and non-responders are summarized in Table 1. The overall response rate (ORR) in this retrospective cohort was (30/69, 43.5%). Fifty percent of the responses occurred within 12 months. The complete resolution rate (CRR) in the whole cohort was (21/69, 30.4%). The mean duration of response was 356 days with 95% CI, 182–432, and the median duration of response was 345 days with 95% CI, 201–525.

In time-to-event Cox regression proportional hazard model analyses, a significant difference in progression was observed between AH and EEC (HR 2.01; 95% 1.039–3.836, LRTP < 0.038) in univariable analysis, but this was not statistically significant in multivariable analysis (HR 1.24; 95% CI 0.984–2.88, LRTP < 0.078) (Table 2). There were 4 women with grade 2 EEC included in this study and the ORR was 2/4 (50%). The ORR in women <50 years was 13/20 (65%). Both age and BMI were associated with treatment response in univariable Cox regression analyses, but both were not statistically significant in multivariable analyses (Table 2). The recurrence rate in women who had responded initially to LNG-IUD treatment but later relapsed was 10/30 (33.3%). The rate of recurrence was higher in EEC (3/7, 42.8%) compared to AH (7/23, 30.4%) and the mean duration to recurrence was 12 months with 95% CI, 9–19 months. There was no significant difference in treatment outcome between LNG-IUD treated compared to those women that had supplementary or maintenance oral progestin (Table 1; Table 2). The study included 20 women treated with metformin as part of their routine diabetic or PCOS regimen and there was no significant difference in the (ORR) between women who received additional metformin treatment and those that did not (HR 0.97;95%CI, 0.467–1.772, LRTP = 0.78) (Table 1; Table 2). There was no statistically significant difference between treatment responders and non-responders when comparing high (>50%) vs. low (≤50%) tumour epithelial PR expression (Fisher’s exact test, *p* = 0.42). However, we noted a significant difference between treatment responders and non-responders in stromal PR expression (Fisher’s exact test *p* < 0. 049). Nevertheless, both stromal and tumour PR expression were not significant in time to event univariable Cox regression analyses (Table 2). When stratified by pre-treatment histologic diagnoses, there was a significant difference in ORR between AH and EEC (64% vs. 23%), *p* < 0.0001, age (*p* < 0.043), and stromal PR expression (*p* < 0.001) (Table S1). BMI, treatment indication, biopsy type (pipelle vs. curette), hysterectomy status, FGFR2 protein expression or FGFR2 isoform and PR tumour expression were not statistically significant (Table S1).

### 2.2. Association of FGFR2 Isoforms with Treatment Outcome

**Figure 3:**
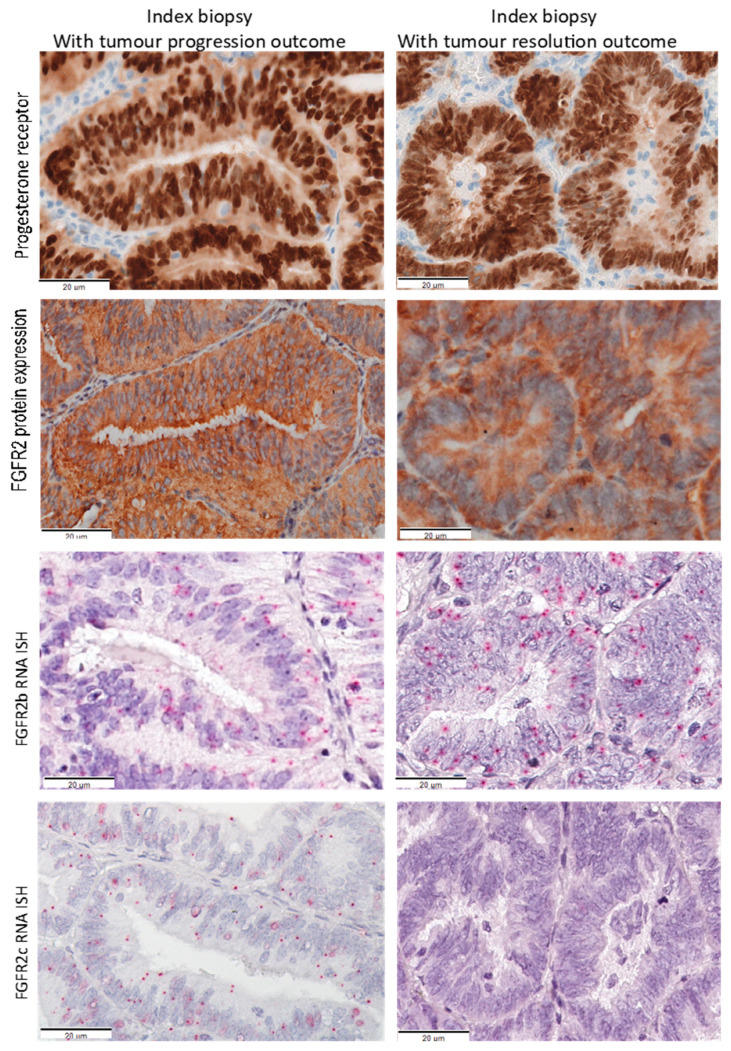
Representative images of PR protein, FGFR2 protein, FGFR2b and FGFR2c mRNA from two different patients with EECs treated with LNG-IUD. Serial sections from the pretreatment diagnostic biopsy of EEC from patient with tumour progression (left panels) and tumour resolution (right panel). Red dots indicate RNA ISH signal product and blue shows nuclear counterstain with haematoxylin. FGFR2c, Fibroblast Growth Factor Receptor 2c isoform; FGFR2b, Fibroblast Growth Factor Receptor 2b isoform; IHC, immunohistochemistry; ISH, In Situ Hybridization; PR, progesterone receptor.

From the 89 samples assessed, FGFR2 isoform status was successfully determined in 72 cases, with 17 cases excluded due to cores missing during BaseScope RNA ISH assessment (n = 10), cores contain stroma only (n = 4) and PPIB housekeeping gene negative (n = 3). Representative images of PR, FGFR2 protein, FGFR2b and FGFR2c expression from serial sections of EEC index biopsies that showed tumour progression and resolution following LNG-IUD treatment is provided in Figure 3.

Exclusive FGFR2b expression (FGFR2b+/FGFR2c-) was documented in 47 tumours, 25 with AH and 22 with EEC (Table 3). Twelve tumours (4 AH, 8 EEC) had co-expression of both isoforms (FGFR2b+/FGFR2c+), and for simplicity are referred to as FGFR2c+. This included two of the cases excluded from outcome analyses due to incomplete clinical follow-up data or insufficient treatment duration (Table 3). Nearly all women with their tumours positive for FGFR2c+ were non-responders 9/10 (90%). In brief, 7 had progression, 2 persistent disease and one woman with an initial response relapsed after 7 months. Notably, loss of expression of both FGFR2 isoforms (FGFR2b-/FGFR2c-) was found in 13 tumour samples (4 of these were excluded from outcome analyses) (Table 3) with loss of both FGFR2 isoforms also associated with progestin treatment failure.

In univariable Cox regression analyses, women with FGFR2c expression were 5-fold more likely to progress (HR 5.08; 95% CI, 2.02–12.77, LRTP < 0.0001) compared to those with exclusive FGFR2b expression. Multivariable Cox regression analyses adjusted for cofounding clinicopathologic parameters (age at diagnosis, BMI, pretreatment histologic diagnosis, tumour grade) was performed. Interestingly, FGFR2c expression (HR 4.5; 95%CI, 1.92–11.32, LRTP < 0.002) was significantly associated with shorter time to progression (Table 2). Age at diagnosis, baseline BMI, pre-treatment histologic diagnosis, and FIGO grade were associated with reduced time to progression in univariable Cox regression analyses but lost their significance in multivariable analyses (Table 2). FGFR2 protein expression was not statistically significant (*p* = 0.378) as the antibody detects both isoforms, highlighting the importance of RNA ISH to determine expression of each isoform.

**Figure 4:**
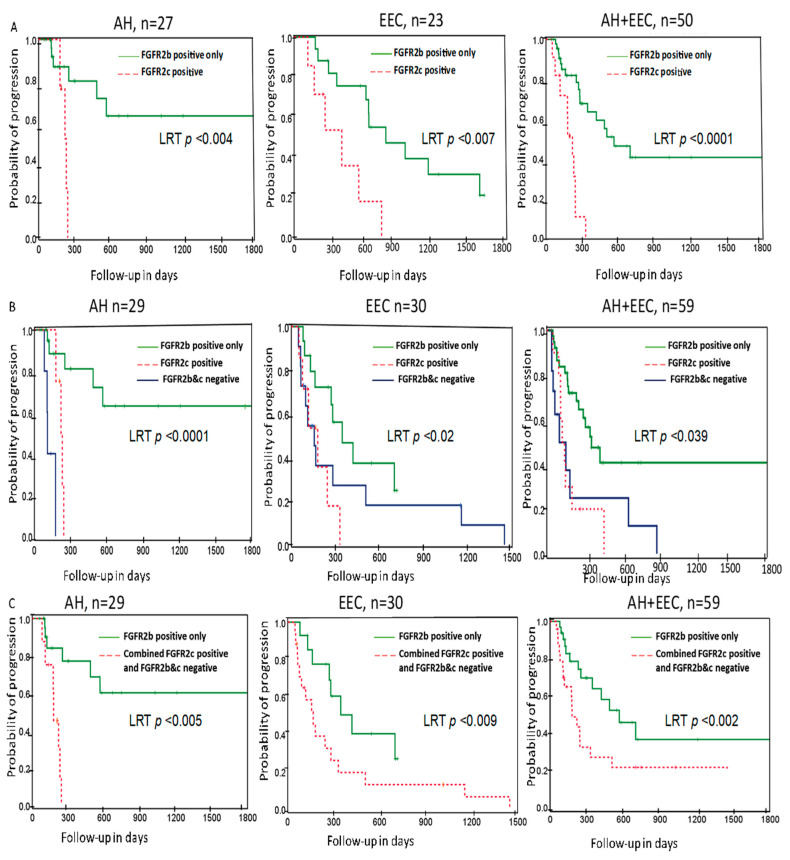
Probability of progression according to the FGFR2 isoform status with and without stratifying by pre-treatment histologic diagnosis in the LNG-IUD treated cohort. Kaplan–Meier curve showing progression probability (**A**) exclusive FGFR2b expression (FGFR2b+/FGFR2c-) vs. FGFR2c positive (FGFR2b+/FGFR2c+) cases. (**B**) exclusive FGFR2b (FGFR2b+/FGFR2c-) vs. FGFR2c positive (FGFR2b+/FGFR2c+) vs. negative for both isoforms (FGFR2b-/FGFR2c-) (**C**) exclusive FGFR2b expression (FGFR2b+/FGFR2c-) vs. FGFR2c positive (FGFR2b+/FGFR2c+) combined with negative for both isoforms (FGFR2b-/FGFR2c-). AH, atypical hyperplasia; EEC, Endometrioid endometrial carcinoma; FGFR2c, Fibroblast Growth Factor Receptor 2c isoform; LRTP, Log Rank Test Probability.

Kaplan–Meier curve analysis was performed to evaluate the association of FGFR2c expression with progestin treatment response. Interestingly, patients with FGFR2c expression had a shorter time to progression (LRTP < 0.0001) overall, and this remained statistically significant when patients were stratified by pre-treatment histologic diagnosis, LRTP < 0.004 and LRTP < 0.007 for AH and EEC, respectively (Figure 4A). We also found loss of expression of both FGFR2 isoforms was significantly associated with shorter time to progression in the whole cohort, as well as when stratified by pretreatment histologic diagnosis (Figure 4B). Hence, Kaplan–Meier curve analyses was performed combining cases with expression of FGFR2c and loss of both FGFR2 isoforms vs. expression of FGFR2b which showed that exclusive FGFR2b expression was significantly associated with longer time to progression (Figure 4C).

## 3. Discussion

Progestin therapy has been used as an alternative to surgical management for AH and early-stage well-differentiated EEC in the setting of fertility preservation and/or presence of comorbidities that prevent surgery. However, the response to progestin-based treatment has been reported to be extremely variable ranging from 23 to 94% [25]. This disparity is partially due to patient heterogeneity, the lack of consensus on duration of adequate treatment, inconsistencies in the methodological approaches used and small sample sizes. Our cohort is based on retrospective data of women with (AH) and endometrioid EC treated with LNG-IUD ± oral progestin from the Mater Hospital. The CRR in our retrospective cohort was ~30%. The ORR across the whole cohort was 44% and women with AH showed a higher ORR (64%) compared to women with EEC (23%). Recent reports of LNG-IUD response rates in AH and EEC in pre- and post-menopausal women have also shown a higher response rate in AH compared to EEC [8,9,10,26]. This suggests progestin treatment efficacy decreases with tumour progression. Maggiore and colleagues reported the highest ORR in EEC (13/16, 81%), however it should be noted that the mean age and BMI of this cohort was 34 years and 25 Kg/m^2^ [10]. In contrast, the mean age of our current cohort was 54 and the median BMI was 48 Kg/m^2^. This is most similar to the mean age (54 years) and BMI (45 Kg/m^2^) reported by Behrouzi and colleagues which was associated with an ORR of 56% [8]. In a recent prospective trial of LNG-IUD treatment a response rate of 89% and 66% was seen at 12 months in patients with AH and EEC, respectively [26]. Although the median age and median BMI was lower in these clinical trial participants (48 y and 45 Kg/m^2^), it is unknown whether these differences in the patient cohort characteristics are responsible for the lower ORR seen in the Mater cohort. The ORR in women <50 years in our cohort was 65% and in older women (>60 years) it was 39% suggesting that response rate does decrease with age. The indication for therapy in the majority of our women was the presence of comorbidities that precluded hysterectomy at time of diagnosis, which differs from the primary aim of fertility preservation reported in studies with a higher response rate. We opted to include the four patients with grade 2 EEC to show the response rate in this cohort (2/4, 50%). When combined with reports of LNG-IUD response rates in other small cohorts of G2 EEC patients [9] [10], an ORR of 11/16 (69%) was seen in patients with G2 EEC.

The recurrence rate of 33% in our cohort is slightly lower compared to the recently reported figure of 41% in AH [11] and EEC [10]. In contrast, a recurrence rate of only 10% was reported in the recent prospective trial with only 12 months follow-up [26]. Notwithstanding the latter result, our data suggests a significant number of patients may relapse and hysterectomy planning is required as a definitive treatment.

The ability to predict which patients might respond best to hormone treatments was identified as one of the top 10 unanswered research questions by patients, carers and health professionals in 2015 [27]. As such, one of the main objectives of this investigation was to assess whether expression of the two major FGFR2 isoforms was associated with LNG-IUD response in women with AH and early-stage EEC. Isoform switching of FGFR2 (expression of the FGFR2c mesenchymal isoform in epithelial cells) was identified in 10/59 patients with detailed clinical follow-up and 9/10 of these patients failed to respond to LNG-IUD treatment (showed persistent disease or progression). The one EEC patient with FGFR2c expression who showed an initial response, recurred after 7 months. Approximately one third of women with AH will progress to endometrial cancer in the absence of treatment [4]. In this cohort, FGFR2c expression was found in 4 women with AH, all of whom subsequently progressed to endometrial cancer despite progestin treatment.

The pattern of the FGFR2c expression in this cohort was different from that previously reported in the Vancouver cohort which was characterized by higher FIGO grade and FIGO stage [24]. In these index biopsies FGFR2c expression is relatively low (2–3 signal dots per cell, Figure 3 left panel), and nearly all FGFR2c positive tumours also co-expressed FGFR2b. This suggests that in AH and well-differentiated early-stage EECs, complete isoform switching from FGFR2b to FGFR2c had not occurred in all tumour cells. We hypothesise that FGFR2c expression could represent subclonal cells undergoing EMT early in disease progression in these patients. Nevertheless, both univariable (HR 5.08, *p* < 0.0001) and multivariable (HR 4.5, *p* < 0.002) Cox regression model analyses revealed that FGFR2c positive women had a shorter time to progression. This indicates even low expression of FGFR2c expression contributes to progestin treatment resistance in a subset of patients. Conversely, tumours with exclusive FGFR2b epithelial isoform expression had a better response to LNG-IUD treatment and thus expression of FGFR2b could be a potential positive predictive marker for LNG-IUD treatment. A study demonstrated that Ishikawa cell line treated in vitro with progesterone upregulates expression of FGFR2b and co-stimulation with progesterone and FGF7 inhibited cell adhesion and growth [28]. Several studies have documented expression of FGFR2b in normal human cyclic endometrium and EC samples [28,29,30]. Other studies also reported that FGFR2b isoform inhibits tumour growth and promotes differentiation and apoptosis in bladder, prostate, and thyroid cancers [23,31,32].

FGFR2 total protein detected using IHC was not associated with LNG-IUD treatment outcome indicating the value of the innovative RNA ISH assay in detection of each FGFR2 isoform and their predictive role for LNG-IUD treatment. RNA ISH has an advantage in detecting alternatively spliced mRNA that do not have isoform-specific antibodies. RNA ISH is also superior to the RT-PCR or RNASeq in revealing the temporal and spatial expression of biomarkers of interest while preserving the morphological context of the cells or tissues [24].

The mechanism underpinning how FGFR2c expression contributes to progestin treatment resistance has not yet been fully investigated and this is a future direction of study for our laboratory. However, in vitro, in vivo, and clinical studies have shown that FGFR2c expression is associated with increasing cancer cell motility, epithelial-mesenchymal transition (EMT) and stemness [33,34]. EMT and stemness are hallmarks of tumour progression and strongly linked to treatment resistance [35]. We propose clonal cells expressing FGFR2c have autocrine receptor activation and downstream activation of the MAPK pathway and therefore are no longer dependent on hormonal signalling for proliferation and tumour progression. This is supported by several studies in breast cancer showing that FGF/FGFR signalling has been implicated in hormone therapy resistance, and treatment resistance is reversed with FGFR inhibitors [36,37]. Whether FGFR2c also signals through the PI3K pathway in early stages of EEC is unknown, but the high frequency of PI3K activation in EECs carrying activating FGFR2 mutations, and the induction of cell death despite constitutive PI3K pathway activation in cell lines carrying FGFR2 activating mutations [38] suggest that FGFR2c exerts its effect primarily through the MAPK pathway.

Despite the wide use of progestin therapy in clinical practice, the mechanism by which progestin treatment induces tumour growth suppression is not fully understood. PR expression has been extensively investigated as a possible predictive biomarker, however there are conflicting results reported. Some studies reported PR-A is associated with poor tumour differentiation and poor response to progestin, in contrast, another independent study reported PR-B is associated with higher grade and poor response to hormone therapy [13]. While we did not find an association between tumour (epithelial) PR expression and response rate, higher stromal PR expression was associated with a higher response rate. However, neither tumour nor stromal PR expression was significant when time to event Cox regression analyses was performed. Several epidemiological clinical studies have reported that unopposed estrogen was the most common risk factor for endometrial hyperplasia and cancer development [2,39]. Janzen and colleagues showed that higher stromal PR expression sensitizes to progestin treatment with a combination of elegant in vivo studies [40]. Our finding supports these findings that higher stromal PR expression counteracts epithelial proliferation by promoting differentiation and stromal decidualization through paracrine signalling.

Although, the number of patients treated with LNG-IUD vs. combination of LNG-IUD +metformin was not equal, the response rate between the two groups was not significantly different. This finding is consistent with a recent report by Acosta-Torres and colleagues who demonstrated addition of metformin to progestin had no superior outcome in AH and EEC patients [41].

The strengths of this study include our robust inclusion and exclusion criteria and relatively large sample size with long-term clinical follow-up. This is also the first report describing the expression of the two FGFR2 isoforms using very specific and sensitive BaseScope RNA ISH assay and their association with LNG-IUD treatment response.

However, there are several limitations to our study. The major limitation is that the cohort is based on retrospective data collected from a single institution, potentially introducing ascertainment bias. Some women were referred to our unit for management following an initial biopsy elsewhere. As we required at least 2 mm of lesional tissue within the block to ensure diagnostic tissue in the constructed TMA, it is possible this inadvertently selected for women with larger initial tumour volumes, potentially affecting the response rate [9]. The cohort was also dominated by obese/morbidly obese and older women, which may have also partly contributed to the low ORR. In addition, follow-up biopsies at 3 or 6 m were not available for 9 women as they underwent a hysterectomy between 3 and 6 months and may have gone on to show a response with longer treatment. Another possible limitation is that in contrast to prospective clinical trials with a defined endpoint (for example, response at 6 or 12 month), we determined the ORR at any time point. This was done with the intention to capture both early and late responders. However, if anything this should have increased the ORR as previous reports indicates response rates increase from 12 to 18 months [25]. The other weakness of the study is not performing power calculation prior to sample collection due to the nature of retrospective data, the different sampling methods used to determine response and the heterogeneity of the patient cohort, although the later does reflect real world clinical practice. For these reasons, care should be taking in interpretation of the findings in this study, with validation in a larger multi-institutional cohort needed.

## 4. Materials and Methods

### 4.1. Patients

The Mater Pathology database was searched using keywords “curette biopsy and adenocarcinoma,” “uterus and atypical hyperplasia,” “uterus and complex atypical hyperplasia,” and “curette and endometrioid” from 2006 to 2018 to identify women diagnosed with AH or EEC who were treated with progestin/LNG-IUD for a minimum of two months, with subsequent follow-up histology. Exclusion criteria included no available pre-treatment (index) biopsy at Mater Pathology, progestin treatment prior to initial biopsy, benign pathology on review, insufficient lesional tissue within the index biopsy and chemotherapy or radiotherapy for another malignancy within the previous 5 years (Figure 2). A total of 89 women were identified, all of whom were treated with LNG-IUD. In addition, 12 women commenced on additional oral progestin based on clinician choice within 3–6-months of LNG-IUD insertion or as maintenance therapy after removal of the LNG-IUD. Twenty-two women had metformin treatment either for their diabetic treatment regimen (n = 16) or for polycystic ovarian syndrome (PCOS) (n = 6). The dose of metformin administrated was 1000–1500 mg/day orally. Patients were investigated to rule out any evidence of myometrial invasion, and local or distant metastasis using standard of care radiologic evaluation prior to treatment commencement. Demographic, clinical data, diagnostic indexed endometrial biopsies/curettage and subsequent follow up biopsies, hysterectomy samples and outcome data were collected. A gynaecologic pathologist reviewed the biopsies and hysterectomy samples. Samples were reported according to WHO 2014 criteria. There were 40 women with AH and 49 with well-differentiated EECs in this cohort. Patients data were recorded from the date of diagnostic (index) biopsy obtained and/or commencement of Progestin treatment (52 gm levonorgestrel IUD insertion) to the date of hysterectomy (if performed) or last date of follow-up in the clinic. Follow up biopsies were taken every 3–6 months either by curettage or Pipelle, at the clinicians’ discretion.

Treatment outcomes were based on the histologic evaluation of the last follow up biopsy or hysterectomy specimen and defined as previously published by Wheeler [42]. In brief, (I) *resolution* if histology indicated normal proliferative or secretory or atrophied/inactive endometrium; (II) *regression (partial response)* if benign hyperplasia without any atypia and with no focal residual AH or EEC; (III) *persistent* if there was no change from the pre-treatment (indexed) biopsy; IV) *progression* if the last sample indicated EEC when the index biopsy was AH or showed increased FIGO grade from G1 to G2/3 or substantial myometrial invasion or extension to cervix at hysterectomy. *Recurrence* was defined if the patient tumour initially showed complete resolution or regression at any time point of the follow-up biopsies, but subsequent biopsies indicated either AH or EEC. The final treatment outcome was dichotomized into ‘non-responders’ if the patient showed a persistent tumour or progression after treatment for not less than 12 weeks and treatment ‘responders’ if the patient showed tumour regression or resolution at any time point. The primary outcome was overall response rate (ORR) at any time point and secondary outcomes were resolution and regression response rates and recurrence rates. The study was planned, designed and reported according to the REMARK guidelines [43].

### 4.2. Tissue Microarray (TMA) Construction

The pre-treatment diagnostic (index curette biopsies) patient samples were identified and two cores from different sites per patient sample were assembled from the original archive FFPE biopsy blocks into two different TMAs with cores measuring 1.0 mm in diameter using a semi-automated TMA constructor (Beecher Instruments) at Mater Pathology. Serial sections from the two sets of TMAs (4 µm thickness tissue sections) were cut for immunohistochemistry (FGFR2 and PR) and RNA in situ hybridization (ISH) assay (PPIB, FGFR2b and FGFR2c) analyses.

### 4.3. Immunohistochemistry Staining

PR immunohistochemistry (IHC) was performed using the automated slide stainer Ventana Benchmark ULTRA (Roche, Australia) with diagnostic anti-PR antibody (Ventana, clone 1E2). Tumour epithelial and stromal PR expression was recorded separately and scored using the H-score method considering the intensity and percentage of positive cells. Finally, PR H-score was dichotomised using a cut-off of 50% and 10% for epithelial tumour compartment and stromal nuclear stains, respectively. FGFR2 protein IHC staining was performed in two sets of TMAs using anti-FGFR2 antibody (Cat# Ab58201, Abcam, Cambridge, UK) with previously optimized and validated manual protocol [24]. The primary anti-FGFR2 antibody targets the C-terminal FGFR2 which recognizes both FGFR2b and FGFR2c isoforms. IHC scoring was performed by two independent scorers using Histologic ‘H-score’ method as previously published by our laboratory [24] and other groups [16].

### 4.4. BaseScope RNA ISH Assay for Detection of FGFR2 Isoforms and Signal Scoring

Recently, we have developed, optimized, and validated a novel bright field chromogenic BaseScope RNA ISH assay to detect mRNA expression of FGFR2b and FGFR2c isoforms. In this study, FGFR2b, FGFR2c and PPIB positive control (housekeeping gene) mRNA expression were evaluated in the TMAs using a previously published protocol [24]. Briefly, a custom designed, human specific, exon 7–8 junction specific probe (BA-Hs-FGFR2-tv2-E7E8) (1ZZ), (NM_022970.3, 1578-1622 bp) to target the FGFR2b isoform and an exon 7–9 junction specific probe (BA-Hs-FGFR2-tv1-E7E9), (NM_000141.4, 1580–1619 bp) to target the FGFR2c isoform were utilized. A PPIB probe targeting a housekeeping gene was used to verify the RNA quality and only tumour cores showing positive PPIB expression were considered in the final statistical outcome analyses. All probes and BaseScope reagents were purchased from Advanced Cell Diagnostic (ACD), Hayward, CA, USA unless otherwise stated. BaseScope RNA ISH in epithelial tumour compartment scoring was performed manually as previously published [24], as well as via an automated approach using Fiji Image J2 (supplementary method S1). In brief, the slides were scanned using Panoramic automated 3D whole slide scanner with CaseViewer version 2.2 (3DHISTECH, Thermo Fisher Scientific, Australia) and images were exported in TIF format. In Fuji Image J2, a region of interest was selected to count both the number of nuclei and mRNA signal products. Finally, the number of signal products obtained was divided by the number of nuclei to determine the mRNA signal product per cell. Final scores were dichotomized as negative if the RNA ISH score was 0 and positive if the RNA ISH score was > 1. The score obtained from automated counting was compared with the manual score and there was high concordance (96.5%) with kappa coefficient of agreement of 0.97.

### 4.5. Statistical Analysis

Study data were collected and managed using REDCap electronic data capture tools hosted at Mater Hospital [44] and final data was exported into SPSS (version 26) and analysed. Chi-squared or Fisher’s exact tests as appropriate for categorical variables were performed to assess the association between clinicopathologic variables, biomarkers analysed, and outcome. For multiple comparisons, Bonferroni method correction adjustment was applied to minimize the family wise error rate (FWER). Scoring agreement between manual and automated method was estimated by percent of concordance and kappa (k) value. Time-to-event analyses were calculated from the date of progestin treatment commencement (LNG-IUD insertion) to the date of hysterectomy or to the date of last follow-up biopsy confirming disease resolution, persistence, progression, or recurrence. Patients were censored either at the time of hysterectomy (if hysterectomy was performed) or at the last follow up. Out of the 89 women initially identified, 20 women were excluded from outcome analyses due to tumour extension to cervix at the time of diagnosis (n = 2), concurrent radio/chemotherapy for non-gynaecologic cancers (n = 2), inconsistent diagnosis after pathology review (n = 2), progesterone treatment prior indexed diagnosis (n = 2), insufficient clinical data and/or short duration of treatment (<12 weeks) (n = 12) as depicted in Figure 2. Moreover, 17 women were removed from biomarker FGFR2 isoform status exploration due to either missing cores in TMA sectioning (n = 10), lack of expression of the PPIB housekeeping gene (n = 3), or the core contained only stroma (n = 4). Note that 7 women that were excluded due to the clinical exclusion criteria were also dropped due to missing FGFR2 data. The Kaplan–Meier curve analysis was used to predict treatment outcome and p-values were calculated using the log-rank test (LRT) probability. Cox regression proportional hazard models were performed to evaluate the predictive value of each variable. On multivariable Cox regression proportional hazard model analysis, FGFR2 isoform status was adjusted for confounding variables including age, BMI, histologic grade, and pretreatment histologic diagnosis. Variables with *p* < 0.10 were included in a multivariate Cox regression proportional hazard model with a stepwise forward method included in the final analysis. Wald test statistics was performed to assess two-sided 95% CIs for ORRs. In the last step, significant variables from the forward selection model (*p* < 0.05) were included in the final Cox regression proportional hazard model. *p* < 0.05 was considered statistically significant (two-tailed tests).

## 5. Conclusions

In summary, the ORR in our cohort, more specifically in women with EEC is lower than the recent reported rates from younger women. FGFR2c expression appears to be an independent predictive biomarker and is strongly associated with LNG-IUD treatment failure in AH and well-differentiated early-stage EEC. FGFR2b expression on the other hand identifies a cohort of patients more likely to respond to LNG-IUD treatment. This interesting finding requires independent validation in a larger cohort of women treated with LNG-IUD.

## Figures and Tables

**Table 1:** Association of clinicopathologic markers and FGFR2 isoform with treatment outcome in the whole cohort.

Clinicopathologic Characteristics		Responders		Non-Responders		*p*-Value ^a^
		n = 30	%	n = 39	%	
Age Category *	<50	13	43%	7	18%	0.068
	50–60	8	27%	18	46%	
	≥60	9	30%	14	36%	
**BMI Category** *	<30	0	0%	4	10%	0.227
	30–40	6	20%	5	13%	
	≥40	21	70%	28	72%	
	Unknown ^b^	3	10%	2	5%	
**Indication of treatment** *	Comorbidities/surgical risk	12	40%	24	62%	0.36
	Patient choice	3	10%	1	3%	
	Preserve fertility	6	20%	4	10%	
	Symptom control until definite hysterectomy	8	27%	9	23%	
	Unknown	1	3%	1	2.6%	
**Hysterectomy status**	No	20	67%	11	28%	**0.001**
	Yes	10	33%	28	71%	
**Biopsy type during response assessment** *	Curette	19	63%	27	69%	0.466
	Hysterectomy	3	10%	6	16%	
	Pipelle	8	27%	6	16%	
**Pre-treatment diagnosis** *	AH	23	77%	13	33%	**0.0001**
	Endometrioid EC	7	23%	26	67%	
**Grade**	Not applicable ^c^	23	77%	13	33%	**0.0001**
	Grade 1	5	17%	24	62%	
	Grade 2	2	7%	2	5%	
**FGFR2 IHC Score**	Low	5	17%	8	21%	0.378
	High	21	70%	26	67%	
	Missing cores ^b^	4	13%	5	13%	
**FGFR2 Isoform status** *	FGFR2b+/FGFR2c−	23	77%	17	44%	**0.005**
	FGFR2b−/FGFR2c−	2	8%	7	18%	
	FGFR2b+/FGFR2c+	1	3%	9	23%	
	Unknown	4	13%	6	15%	
**Stroma1 PR Score**	≤10%	11	37%	21	54%	**0.049**
	>10%	15	50%	13	33%	
	Missing cores ^b^	4	13	5	13%	
**Epithelial Tumour PR Score**	≤50%	1	3%	3	8%	0.42
	>50%	28	93.4%	33	85%	
	Missing cores ^b^	1	3.3%	3	8%	
**Concurrent Metformin**	LNG-IUD	23	77%	25	64%	0.403
	LNG-IUD + Metformin	7	23%	13	33%	
	Unknown ^b^	0	0%	1	3%	
**Route of progestin therapy**	LNG-IUD only	25	83%	32	82%	0.93
	LNG-IUD + Oral Progestin	5	17%	7	18%	

^a^*p*-value was calculated using Chi-X^2^ test or Fisher’s exact test for dichotomous variables. ^b^ Missing values were not included in *p*-value determination. * multiple comparisons were corrected using the Bonferroni method. *p*-values < 0.05 are indicated in bold. ^c^ FIGO grading only performed in EEC. BMI, Body Mass Index; AH, Atypical Hyperplasia; EEC, Endometrioid Endometrial Cancer; FGFR2, Fibroblast Growth Factor Receptor 2; IHC, Immunohistochemistry; ISH, In situ Hybridization; LNG-IUD, Levonorgestrel Intrauterine device; NA Not applicable; P, Probability; PR, Progesterone Receptor.

**Table 2:** Univariable and Multivariable Cox regression proportional hazard model analyses.

Variables [Reference]	Univariable Analyses				Multivariable Analyses		
	N Total	HR	95%CI	LRTP	HR	95%CI	LRTP
Age in years	69			**0.028**			0.188
Age 50–60 [<50]		2.67	1.163–6.137	**0.021**	1.02	0.99–2.09	0.435
Age ≥ 60 [<50]		3.19	1.319–7.726	**0.01**	1.45	0872–2.65	0.165
BMI 30 (Kg/m^2^)	64			**0.001**			0.052
BMI 30–40 [<30]		1.32	1.15–2.452	**0.002**	1.13	0.84–1.63	0.056
BMI ≥ 40 [<30]		1.17	1.61–3.444	**0.001**	1.37	0.87–1.81	0.29
Pre-treatment diagnosis EEC [AH]	69	2.01	1.039–3.836	**0.038**	1.24	0.984 -2.88	0.078
Grade at diagnosis	69			0.052			0.232
Grade 1 [AH]		1.98	1.023–3.834	**0.043**	1.61	0.86–3.01	0.14
Grade 2 [AH]		2.24	0.499–10.078	0.292	1.54	0.987–5.3	0.097
FGFR2b+/FGFR2c+ [FGFR2b+/FGFR2c- ^a^]	50 *	5.08	2.018–12.774	**0.0001**	4.50	1.92–11.32	**0.002**
FGFR2 protein H-Score High [Low]	60	0.83	0.406–1.684	0.6	-	-	-
PR tumour expression ≥50% [<50%]	63	0.78	0.245–1.367	0.121	-	-	-
PR Stromal expression ≥10% [<10%]	63	0.69	0.346–1.356	0.278	-	-	-
LNG-IUD+ Metformin [LNG-IUD only]	62	0.97	0.467–1.772	0.78	-	-	-
LNG_IUD +oral progestin [LNG_IUD only]	68	0.61	0.268–1.387	0.238	-	-	-

^a^ exclusive FGFR2b (FGFR2b+/FGFR2c-) used as reference. * Nine tumours with no FGFR2 expression were excluded from multivariable analyses. p-values < 0.05 are indicated in bold. Abbreviations: BL, Borderline tumour; BMI, body mass index: CI, confidence interval; AH, atypical hyperplasia; EEC, endometrioid endometrial cancer; HR, Hazard Ratio; FGFR2, Fibroblast Growth Factor Receptor 2; FIGO, International Federation for Gynaecologic Oncology; ISH, In Situ Hybridization; LNG-IUD, Levonorgestrel Intrauterine device; LRTP, Log Rank Test Probability.

**Table 3:** Association of clinicopathologic parameters and outcome with FGFR2 isoform status in 89 women.

Clinicopathologic Characteristics		FGFR2b+ /FGFR2c-		FGFR2b-/ FGFR2c-		FGFR2b+ /FGFR2c+		Unknown Status	
		n	%	n	%	n	%	n	%
Age in years	<50	13	28%	4	31%	3	25%	7	41%
	50–60	15	32%	7	54%	4	33%	6	35%
	>60	19	40%	2	15%	5	42%	4	24%
**BMI in Kg/m^2^**	<30	3	6%	2	15%	2	16.7%	0	0%
	30–40	6	13%	1	8%	3	25%	6	35%
	>40	33	70%	9	69%	6	50%	10	59%
	Missing	5	11%	1	8%	1	8%	1	6%
**Histologic** **diagnosis**	AH	27	57%	4	31%	4	33%	5	29%
	Endometrioid EC	20	43%	9	69%	8	67%	12	71%
**FGFR2 IHC Score**	Low	9	19%	6	46%	2	17%	0	0%
	High	36	77%	7	54%	10	83%	4	23.5%
	missing	2	4%	0	0%	0	0%	13	77%
**PR score stroma1**	≤10	23	49%	8	62%	8	67%	3	19%
	>10	19	40%	4	31%	4	33%	6	36%
	Missing	5	11%	1	8%	0	0%	7	44%
**Tumour PR Score**	≤50%	3	6%	2	15%	1	8%	1	6%
	>50%	41	87%	11	85%	11	92%	10	59%
	missing	3	6%	0	0%	0	0%	6	35%
**Combination** **treatment**	LNG-IUD only	30	71%	10	77%	7	64%	9	56%
	LNG-IUD + Metformin	12	29%	3	23%	4	36%	7	44%
	Missing	0	0%	0	0%	0	0%	0	0%
**Route of treatment**	LNG-IUD only	40	84%	11	85%	11	92%	14	82%
	LNG-IUD + Oral Progestin	7	16%	2	15%	1	8%	0	0%
	Unknown	0	0%	0	0%	0	0%	3	18%
**Treatment** **Outcome**	Responders	23	49%	2	15%	1	8%	4	24%
	Non-responders	17	36%	7	54%	9	75%	6	35%
	Excluded from analyses	7	15%	4	31%	2	17%	7	41%
**Recurrence**	No	31	64%	9	69%	9	75%	10	59%
	Yes	9	21%	0	0%	1	8%	0	0%
	Excluded from analyses	7	15%	4	30.8%	2	17%	7	41%

AH, atypical hyperplasia; BMI, body mass index: CI, confidence interval; EEC, endometrioid endometrial carcinoma FGFR2c, Fibroblast Growth Factor Receptor 2c isoform; FGFR2b, Fibroblast Growth Factor Receptor 2b isoform; IHC, immunohistochemistry; ISH, In Situ Hybridization; LNG-IUD, Levonorgestrel Intrauterine device; PR, progesterone receptor.

## Data Availability

There is no additional data related to this manuscript and crude data used to generate the presented results can be obtained from corresponding author on reasonable request.

## Supplementary Materials

The following are available online at https://www.mdpi.com/article/10.3390/cancers13071703/s1, Table S1. Clinicopathologic characteristics of the cohort stratified by pretreatment histologic diagnosis in the whole cohort. Supplementary method S1 Automated RNA ISH counting method with representative images. The detail methodology for automated determination of the FGFR2 isoforms RNA ISH signal determination is provided as supplementary method S1.
