# Supplementary material for: Fibroblast Growth Factor Receptor 2 Isoforms Detected via Novel RNA ISH as Predictive Biomarkers for Progestin Therapy in Atypical Hyperplasia and Low-Grade Endometrial Cancer
Source: Cancers (Basel). 2021 Apr 3;13(7):1703. doi: 10.3390/cancers13071703 (PMC8038411; doi:10.3390/cancers13071703)
Supplement: Supplementary file 1 [file cancers-13-01703-s001.pdf]

Supplementary Materials

Fibroblast Growth Factor Receptor 2 Isoforms Detected via Novel RNA ISH as Predictive Biomarkers for Progestin Therapy in Atypical Hyperplasia and Low-Grade Endometrial Cancer

Asmerom T. Sengal, Deborah Smith, Rebecca Rogers, Cameron E. Snell, Elizabeth D. Williams and Pamela M. Pollock

Table S1. Clinicopathologic characteristics of the cohort stratified by pretreatment histologic diagnosis in the whole cohort.

Clinicopathologic and Molecular Biomarkers		AH		Endometrioid EC		*p*-value [a]
		n = 36	%	*n* = 33	%	
Age in years *	<50	15	41.7%	5	15.2%	**0.043**
	50–60	10	27.8%	16	48.5%	
	>60	11	30.6%	12	36.4%	
BMI Kg/m[2] *	<30	1	2.8%	3	9.1%	0718
	30–40	6	16.7%	5	15.2%	
	>40	26	72.2%	23	69.7%	
	Unknown [b]	3	8.3%	2	6.1%	
Indication for treatment *	Comorbidities/surgical risk	15	41.7%	21	63.6%	0.413
	Patient choice	2	5.6%	2	6.1%	
	Preserve fertility	7	19.4%	3	9.1%	
	Symptom control awaiting surgery	11	30.6%	6	18.2%	
	Unknown [b]	1	2.8%	1	3%	
Biopsy type at time of response assessment *	Curette	24	66.7%	22	66.7%	0.967
	Hysterectomy	5	13.9%	4	12.1%	
	Pipelle	7	19.4%	7	21.2%	
Hysterectomy status	No	19	52.8%	12	36.4%	0.068
	Yes	17	47.2%	21	63.6%	
FGFR2 protein IHC Score	Low	8	22.2%	5	15.2%	.512
	High	25	69.4%	22	66.7%	
	Missing cores [b]	3	8.3%	6	18.2%	
FGFR2 Isoform Status *	FGFR2b+/FGFR2c−	23	63.9%	17	51.5%	0.078
	FGFR2b−/FGFR2c−	4	11.1%	5	15.2%	
	FGFR2b+/FGFR2c+	4	11.1%	6	18.2%	
	Unknown [b]	5	13.9%	5	15.2%	
PR H-score stroma	≤10%	10	27.8%	22	68.8%	**0.001**
	>10%	22	61.1%	6	18.8%	
	Missing cores	4	11.1%	4	12.5%	
PR H-score in tumour compartment	≤50%	1	2.8%	3	9.1%	0.523
	>50%	32	88.9%	27	81.8%	
	Missing cores [b]	3	8.3%	3	9.1%	
Combination treatment	LNG-IUD only	28	77.1%	21	63.6%	0.222
	LNG-IUD + Metformin	8	22.9%	12	36.4%	
Route of treatment	LNG-IUD only	29	80.5%	28	84.8%	0.60
	LNG-IUD + Oral Progestin	7	19.5%	5	15.2%	
Treatment Outcome *	Resolution	16	44.4%	5	15.2%	**0.0001**
	Regression	7	19.4%	2	6.1%	
	Persistent	6	16.7%	11	33.3%	
	Progression	7	19.4%	15	45.5%	
Recurrence status	No	29	80.6%	30	90.9%	0.222
	Yes	7	19.4%	3	9.1%	

[a] *p*-value was determined using Chi-X^2 test; * multiple comparisons were corrected using Bonferroni test. [b] Missing parameters from respective clinicopathologic characteristics that were not considered in *p*-value determination. *p*-values <0.05

are in bold. Abbreviations: AH, atypical hyperplasia; BMI, body mass index: CI, confidence interval; EEC, endometrioid endometrial carcinoma; HR, Hazard Ratio; FGFR2, Fibroblast Growth Factor Receptor 2; LNG-IUD, Levonorgestrel Intra-uterine device.

Supplementary method S1. Automated RNA ISH signal counting

Protocol for counting BaseScope RNA ISH signal product using Fiji Image J2 with representative images

A) Cell/nuclei counting

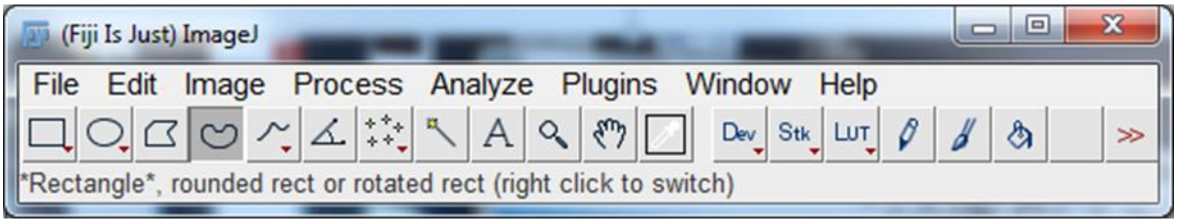

1. From the Fiji ImageJ2 tabs, select **File** > **Open.**

2. Select the image you want and click **Open**.

3. Find a region of interest in your image where you can select a nuclear stain and a probe stain.

4. Zoom into your region of interest using the magnifying glass tool.

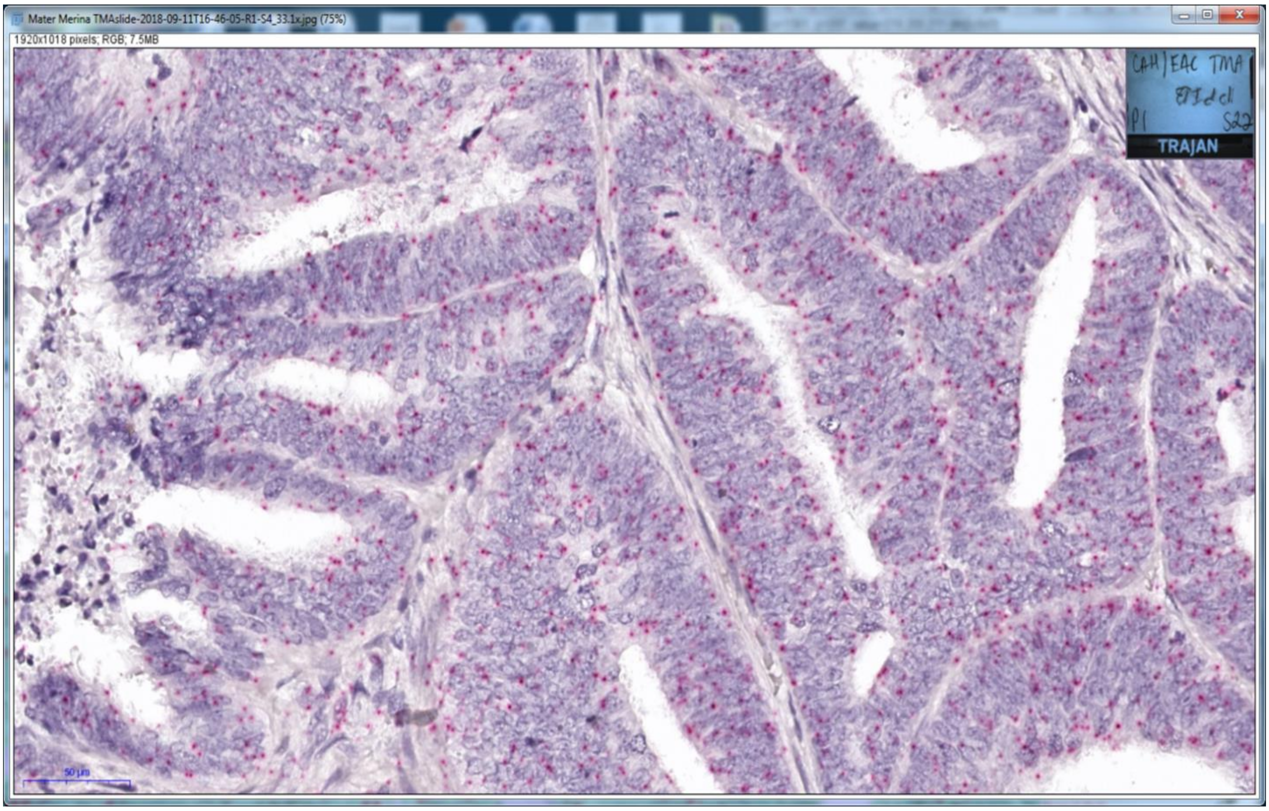

5. Select the rectangle tool.

6. From the ImageJ tabs, select Image > Colour > Colour Deconvolution.

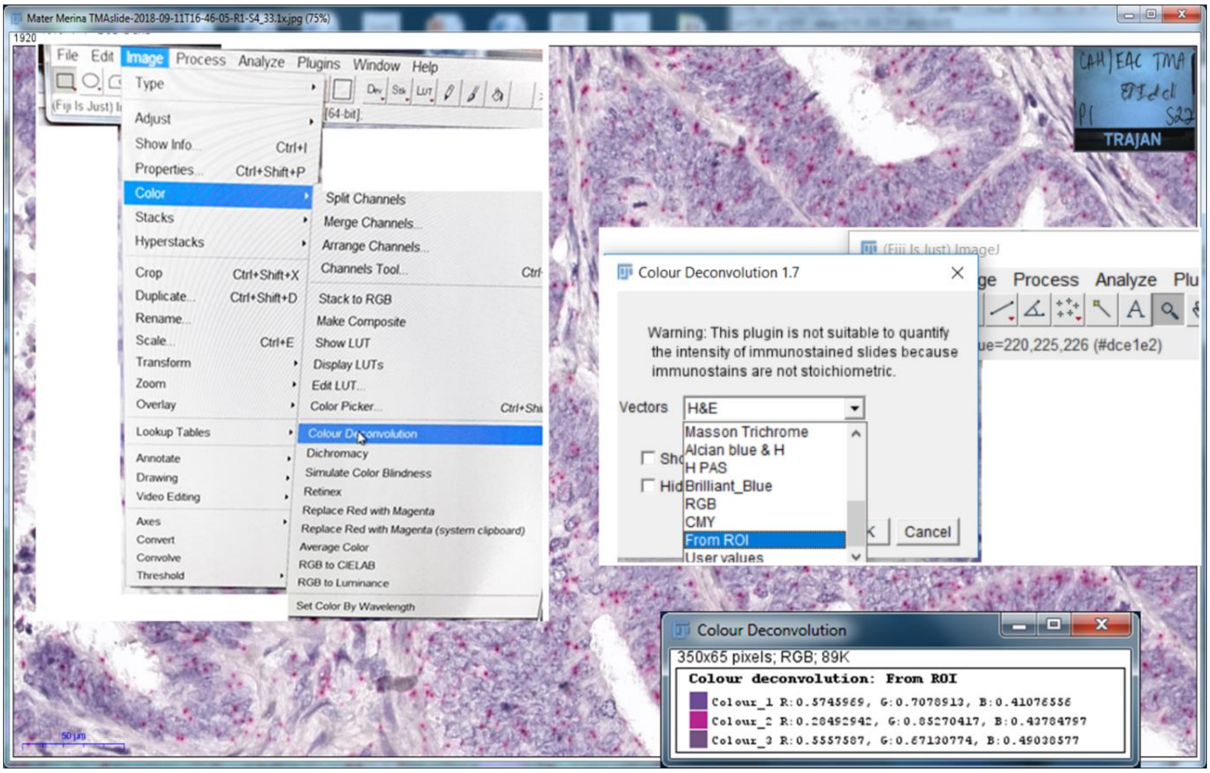

7. From the options menu, select from region of interest (ROI).

8. Using the rectangle tool, do the following in the image:

I. Select an example of a nucleus stain for colour 1.

Right-click to move onto the next step

II. Select an example of a probe stain for colour 2.

Right-click to move onto the next step

III. Select an example of a background stain for colour 3.

Right-click to move onto the next step

Run the colour deconvolution.

You should see an output of three images, one for each channel. The first channel will contain the nuclei, the second image the probe (RNA ISH signal)

9 Save these images for further channel determination

Chanel 1 Nuclei (hematoxylin)

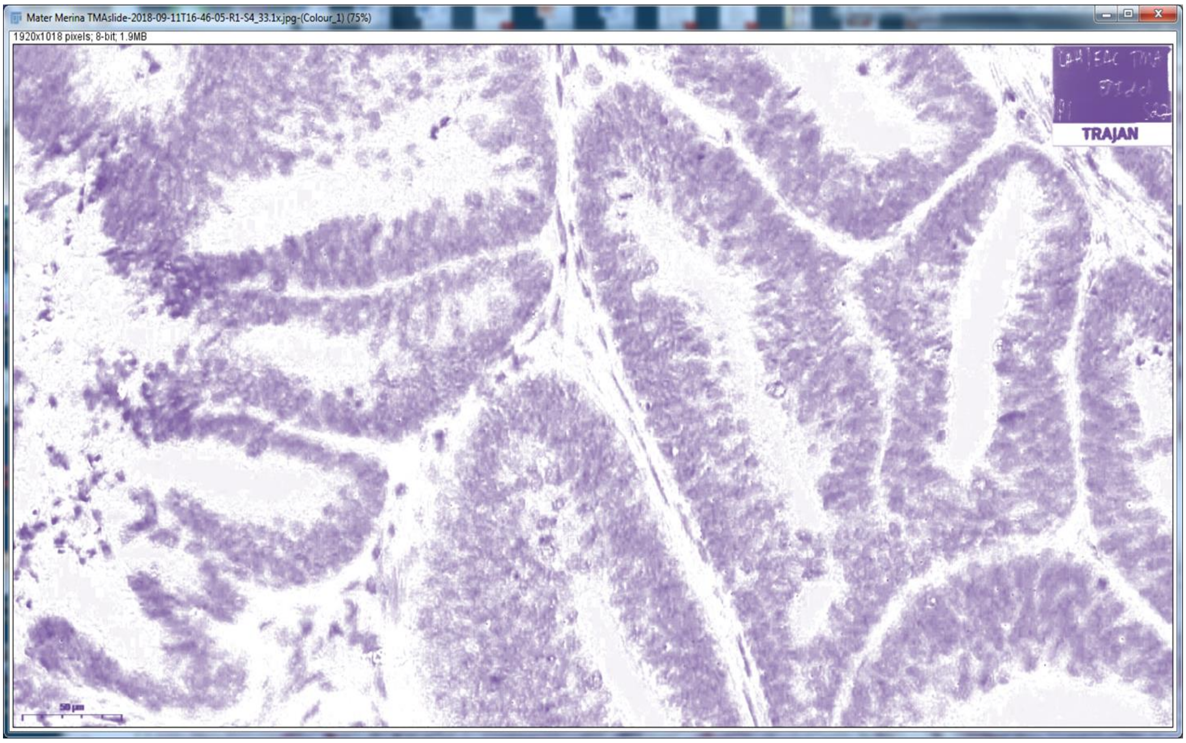

Chanel 2 BaseScope RNA ISH signal **Red**

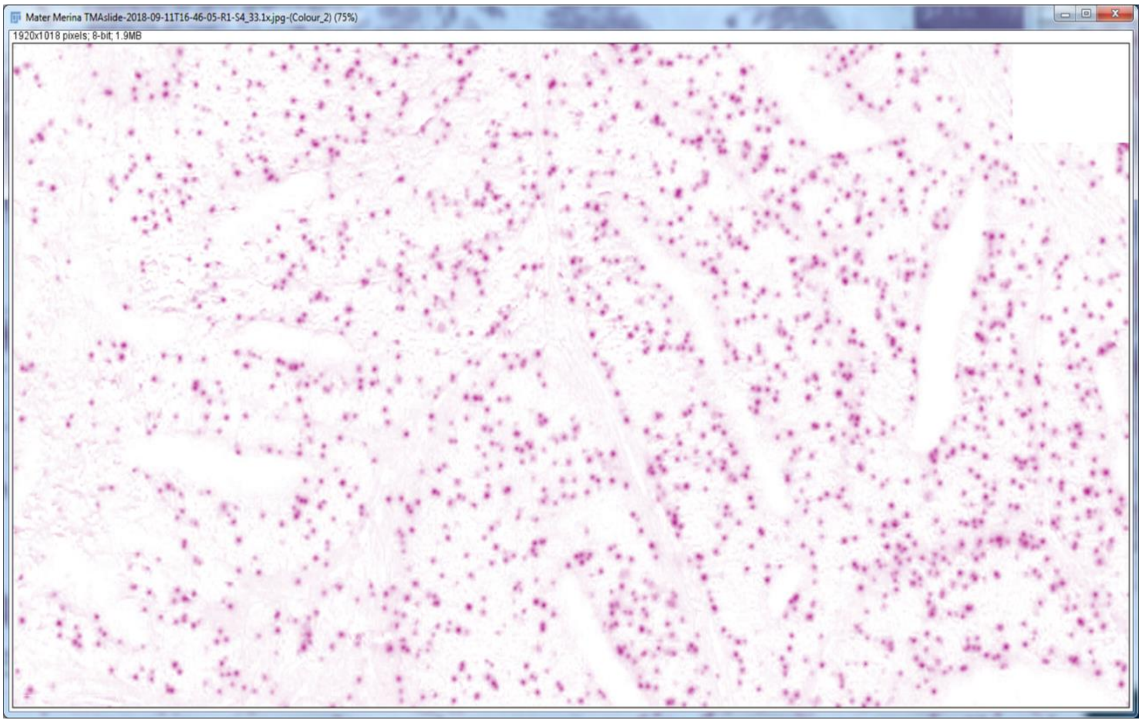

Chanel 3 Background brown

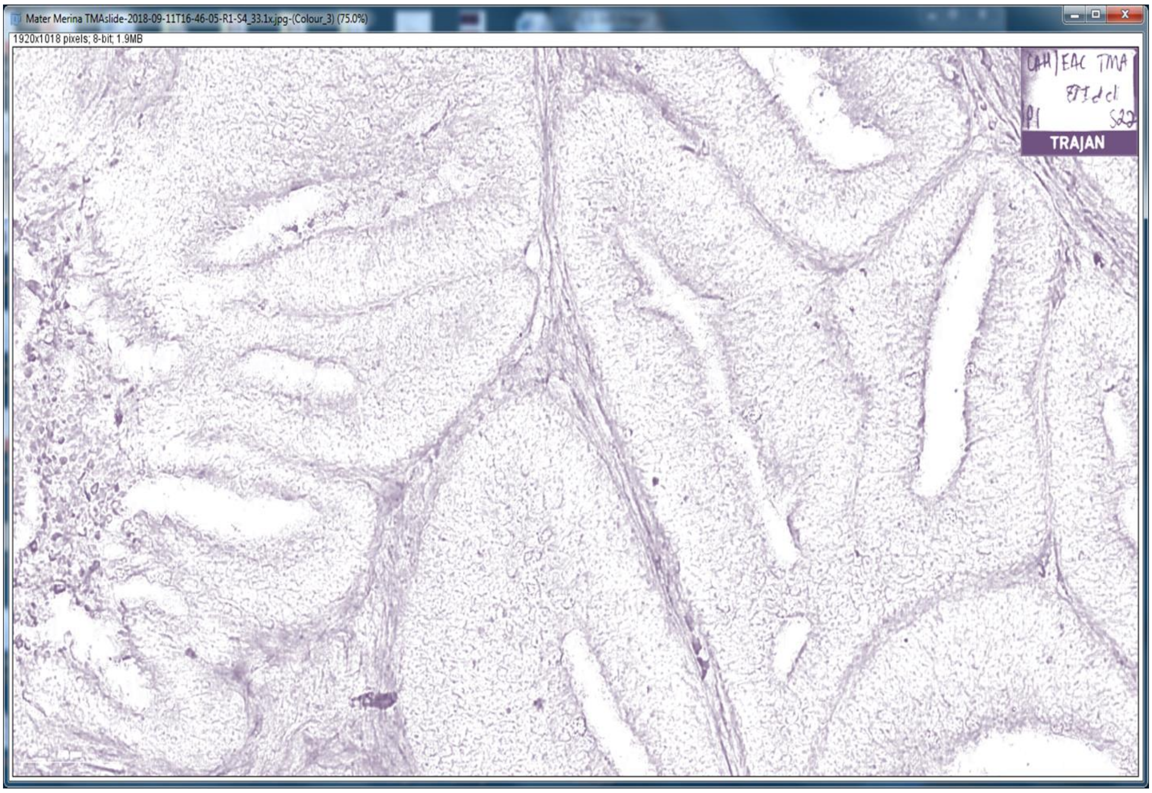

10. To automatically select a threshold based on the two-pixel populations in the image (nuclei and background figure channel 1 above) then select Image > Adjust > Threshold and choose Otsu from the threshold options.

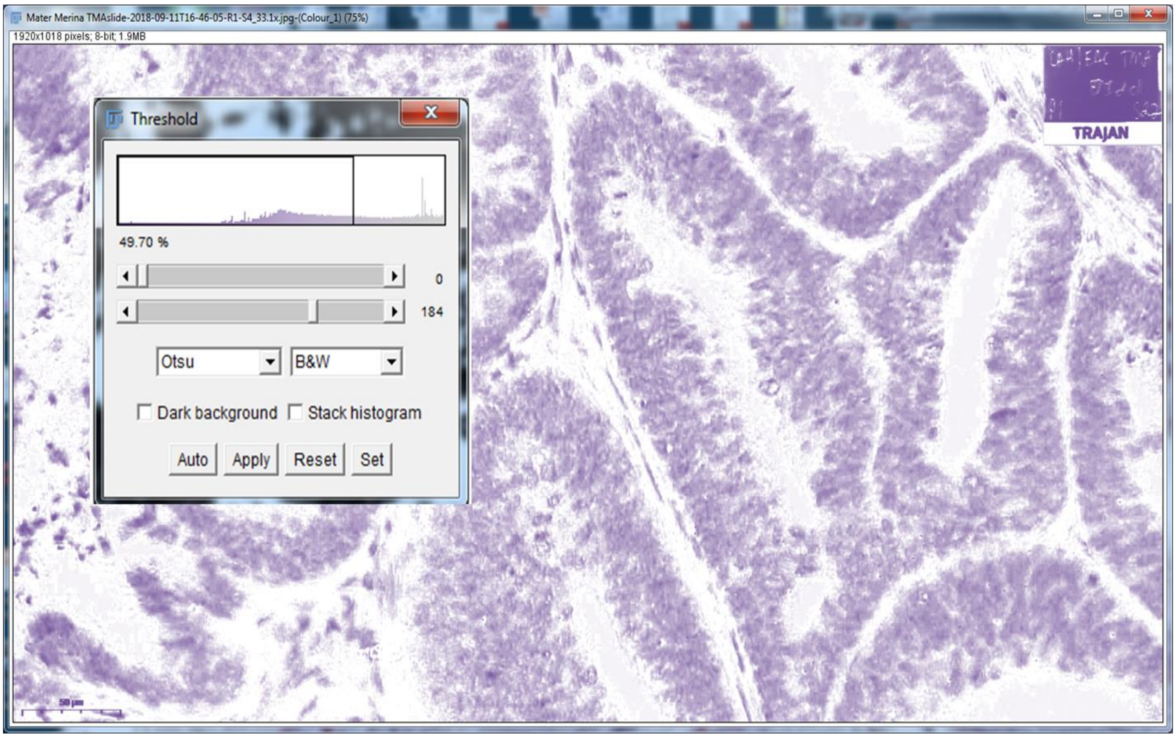

11. Click Apply. See the following images for results (nuclei are shown in black):

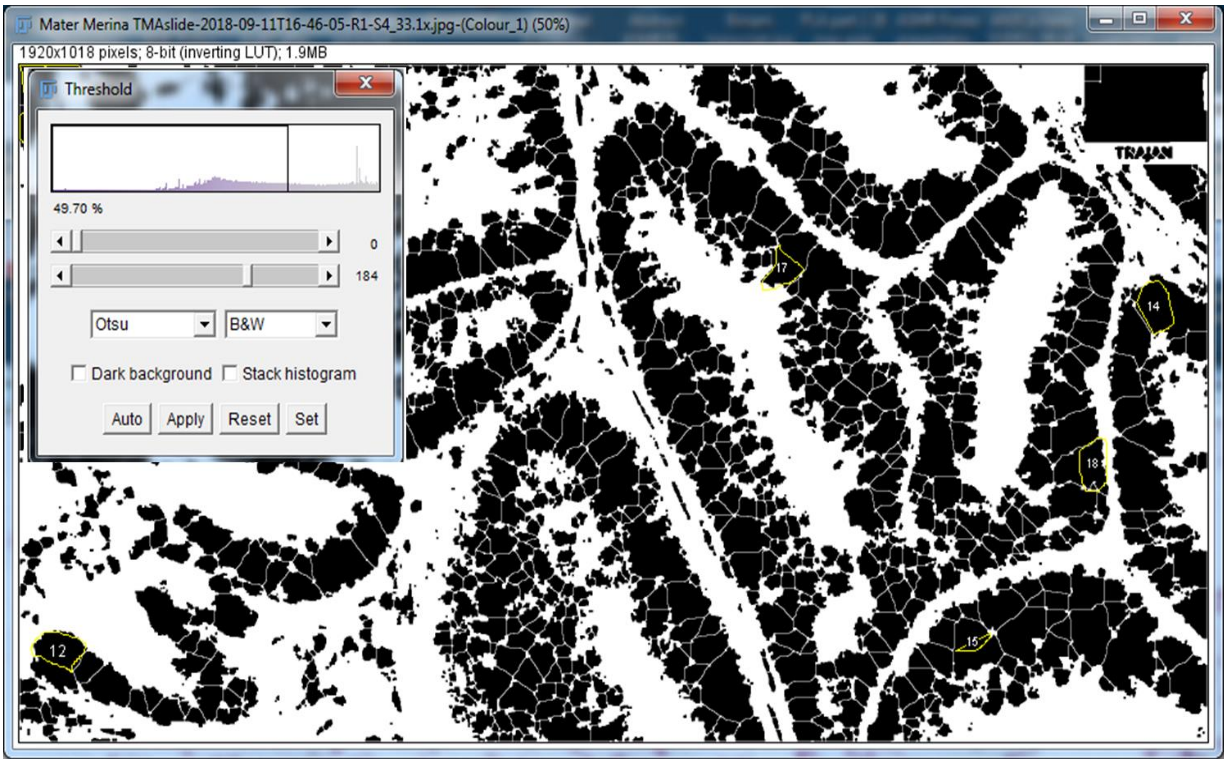

12. If needed, adjust the image to make the nuclei smoother and remove background noise. Use the following morphological operations:

Process > Binary > Close then Process > Binary > Open

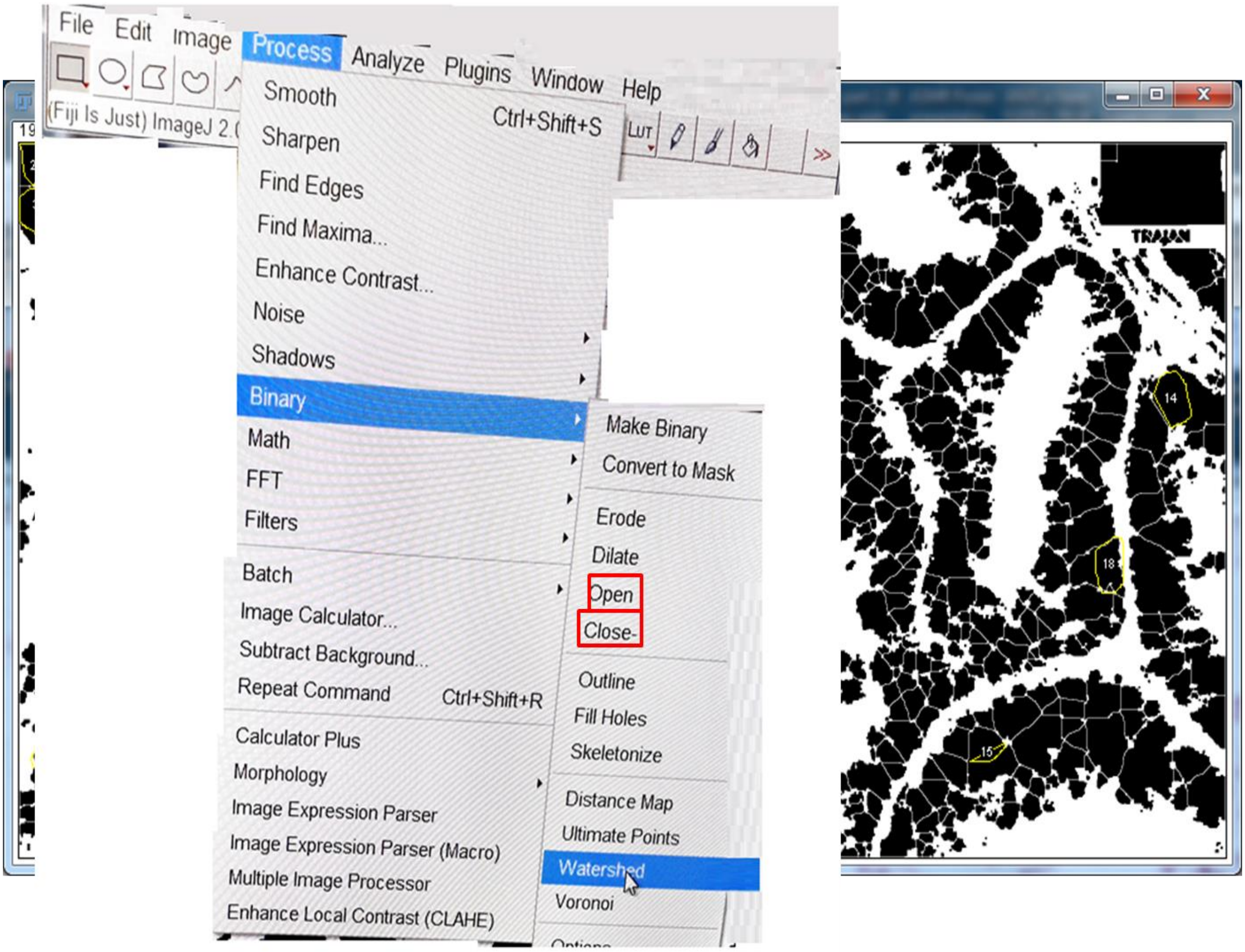

13. To split overlapping nuclei, use Process > Binary > Watershed. See the above image for results:

14. To count the cells, use Analyze > Analyze Particles.

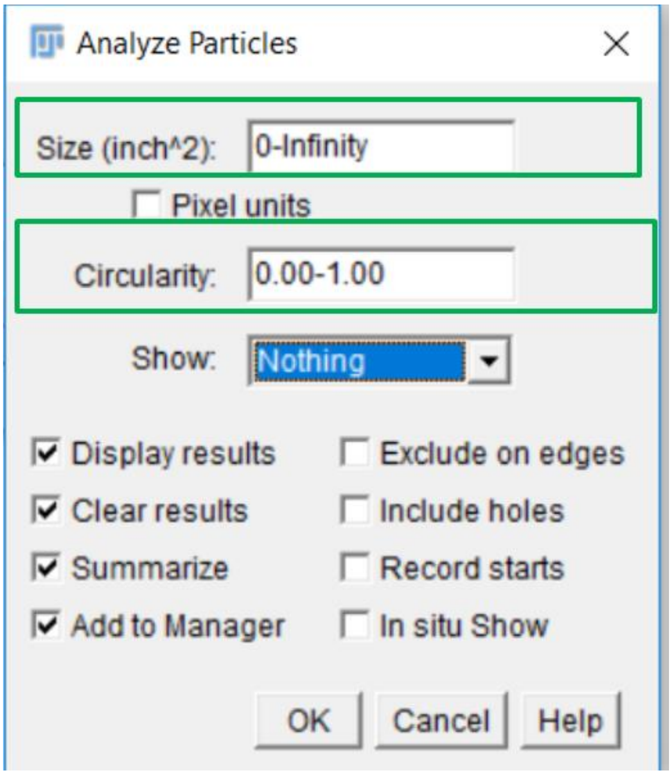

15. Adjust the size and circularity options to avoid counting fragments and to count cells only.

The number of detected cells in the image example is 585.

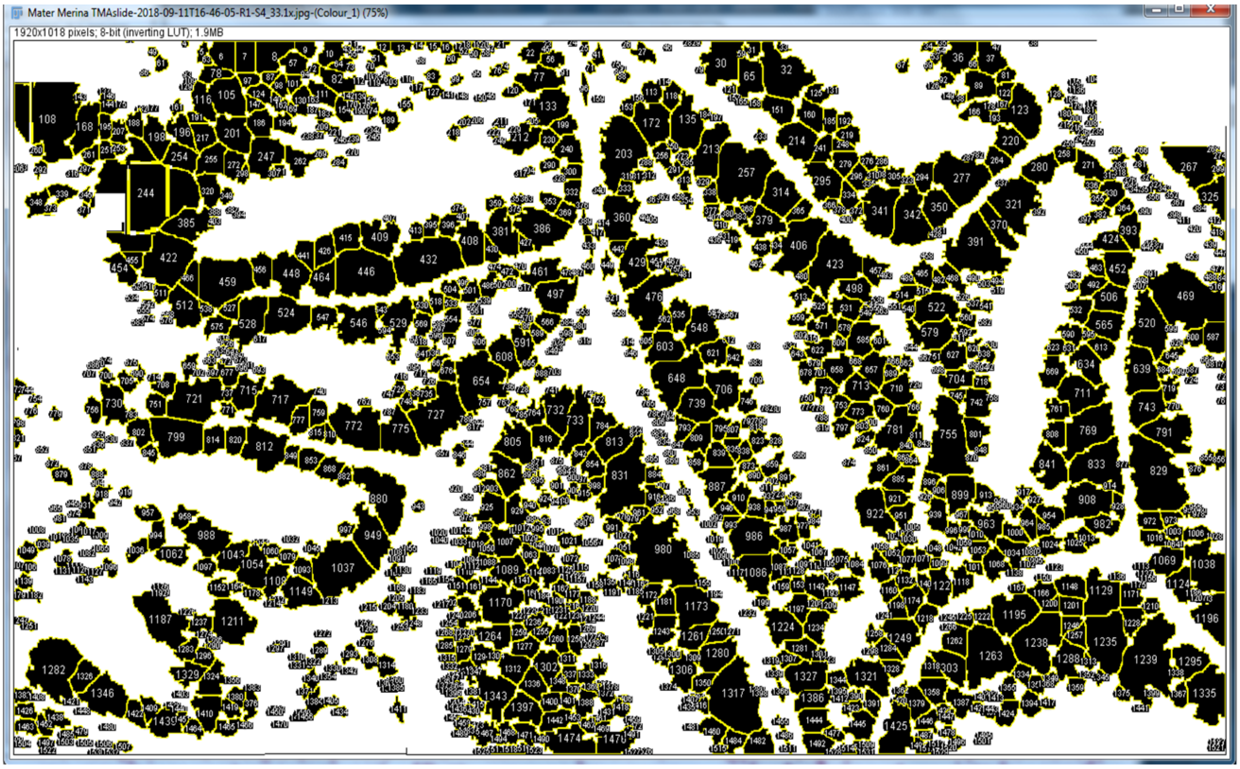

	Area	Mean	StdDev	Mode	Min	Max	IntDen	Median
577	544	255	0	255	255	255	138720	255
578	458	255	0	255	255	255	116790	255
579	639	255	0	255	255	255	162945	255
580	447	255	0	255	255	255	113985	255
581	688	255	0	255	255	255	175440	255
582	383	255	0	255	255	255	97665	255
583	302	255	0	255	255	255	77010	255
584	306	255	0	255	255	255	78030	255
585	343	255	0	255	255	255	87465	255

The total nuclei count in this slide are 585

B) Quantifying the BaseScope RNA ISH Probe signal

To analyse the probe signal, use Weka classifiers.

1. Open the original image and select Plugins > Segmentation > Trainable Weka Segmentation.

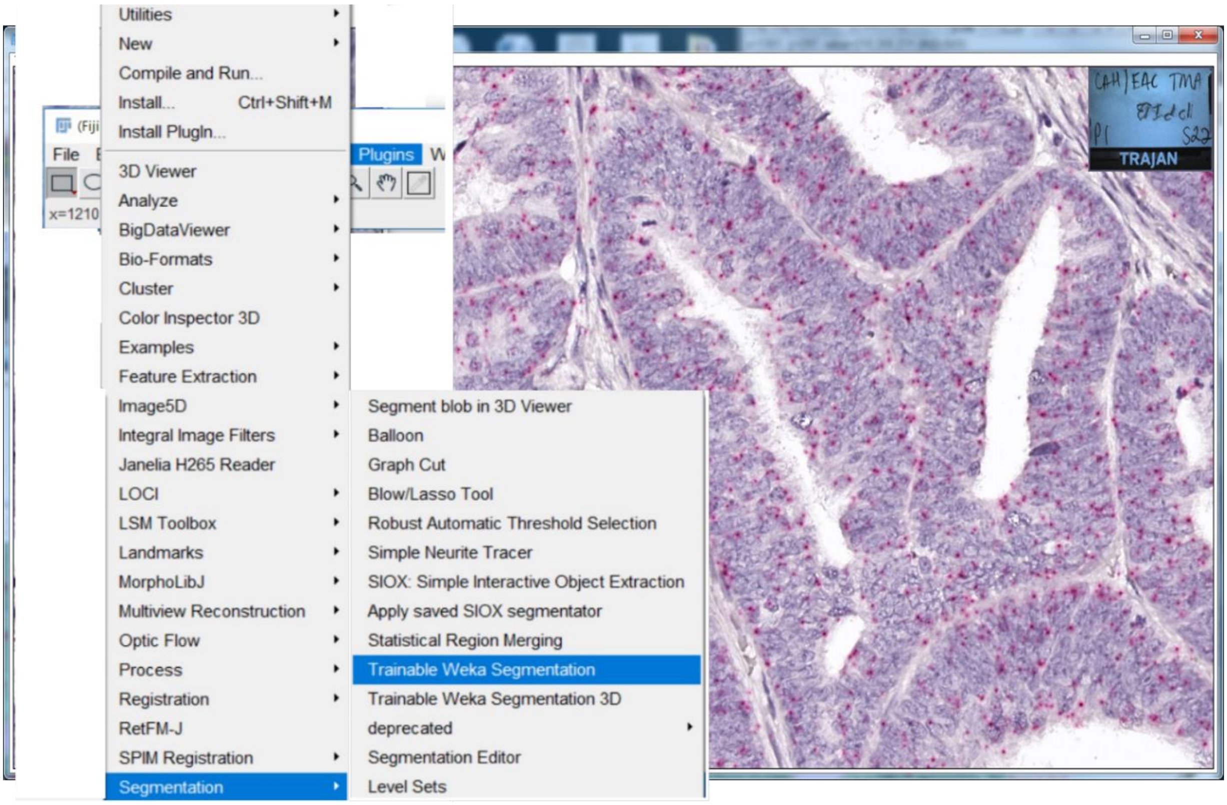

Note that default classes are two

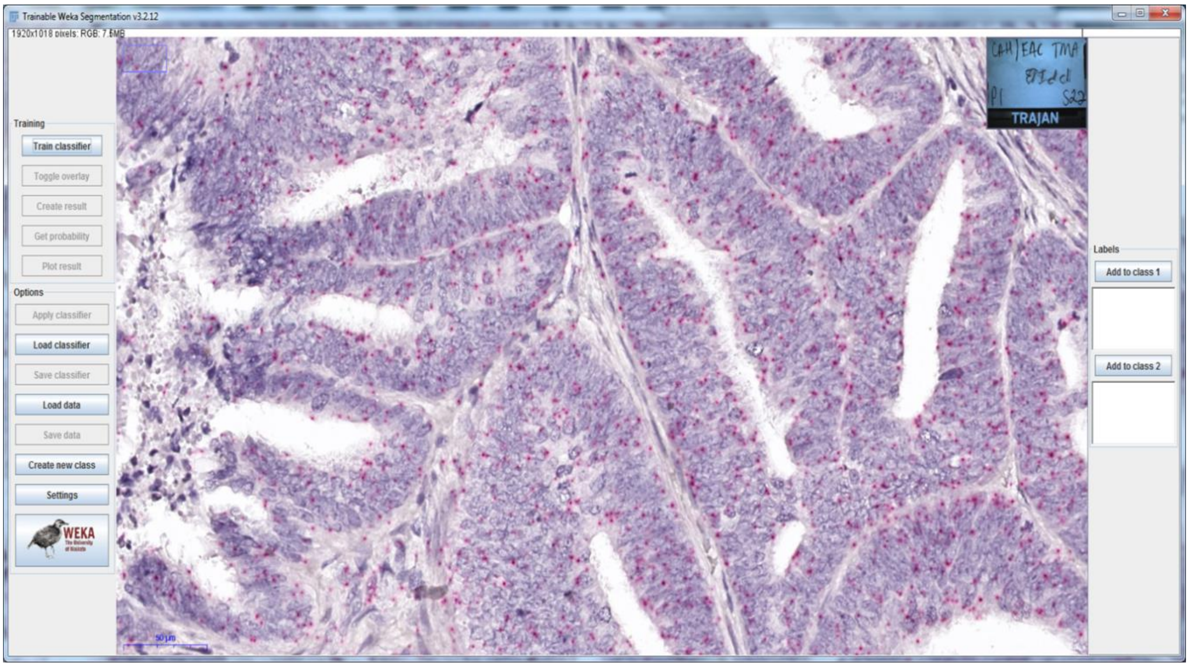

2. Depending on the number of probes you have, add more classifiers by selecting Create new class you can create several classes

The example image has three classifiers: background, nuclei, and probe.

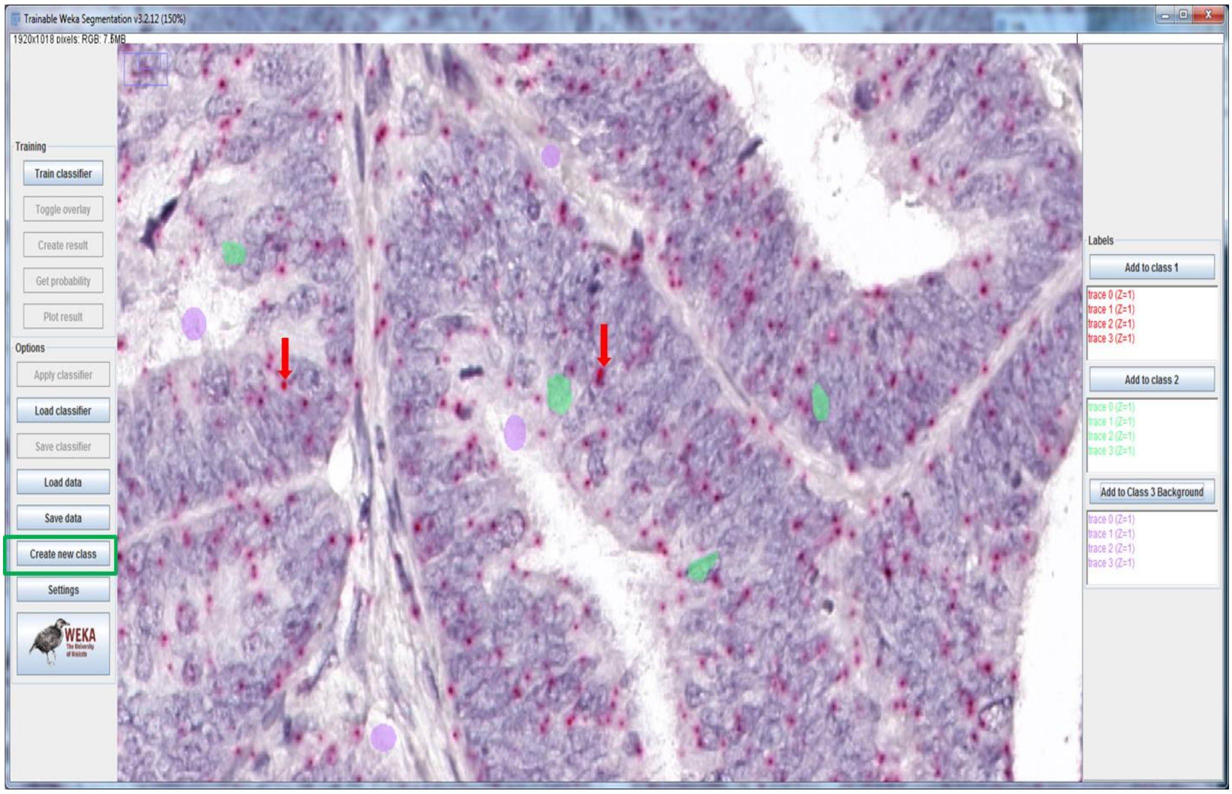

Item 1 Red (indicated with arrow) RNA ISH signal, item 2 Green nuclei and item 3 purple background

3. Using the freehand selection tool, select 2–4 examples for each class as shown. Red arrow indicate probe selected, Green nuclei and pink purple background

4 Select setting

5. Select the following features and click OK.

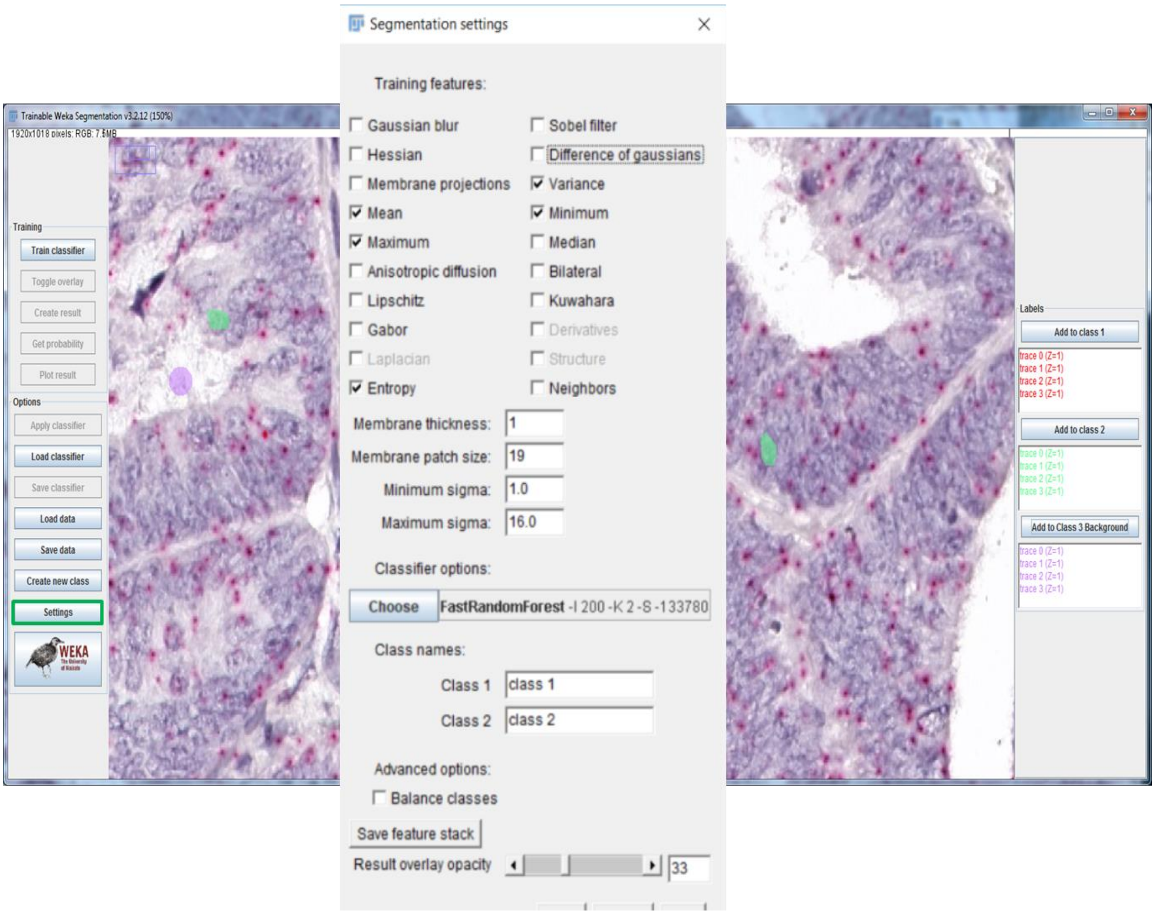

6. Click "Train Classifier" on the main screen and check the results.

Note: This step may take a few minutes depending on the size of your image and computer specification.

7. If the results need refining, select more training regions and repeat steps 4–6.

You should see a result similar to the following image:

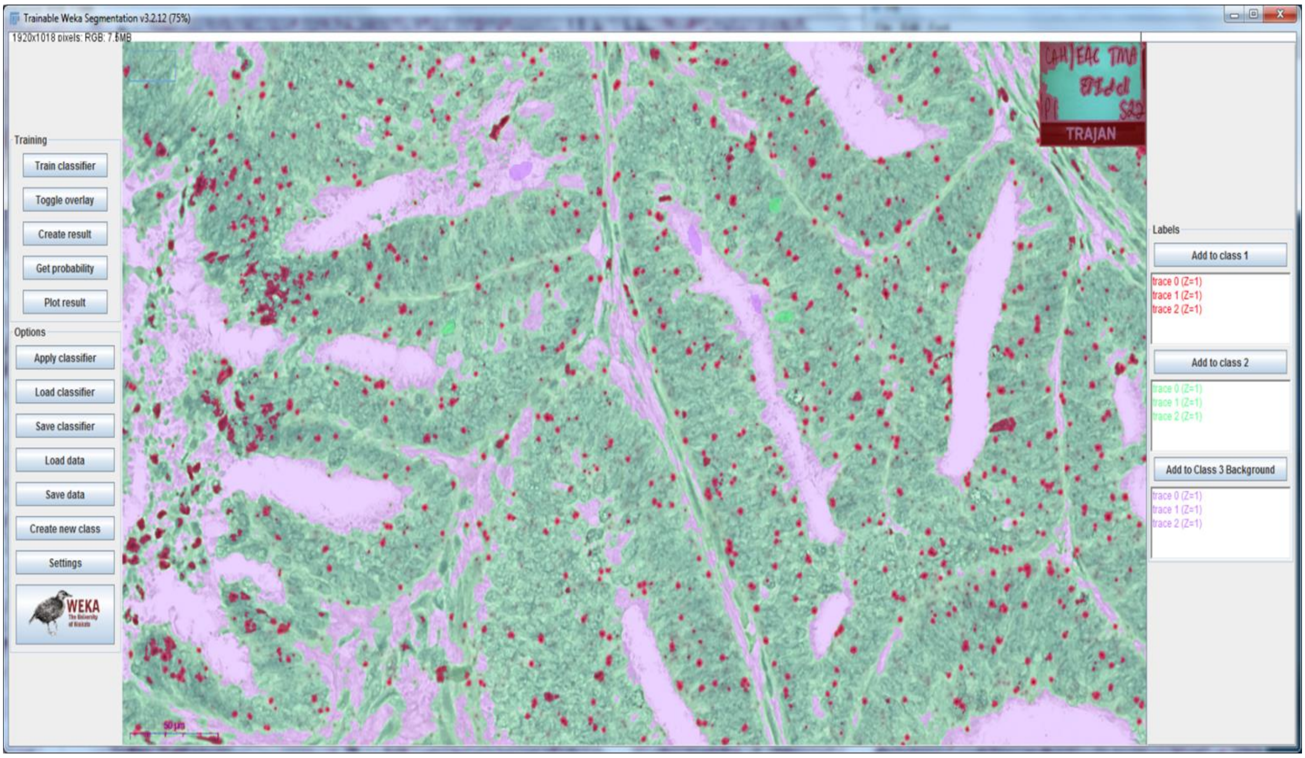

8. Click Create result.

9. Separate the probe class from the other classes:

I. Select Image > Adjust > Threshold.

II. Change the range until only the probe class is visible in the image.

III. Click Apply and close the window.

10. To count the probe spots:

I. Select Analyze > Analyze Particles.

II. Adjust the circularity and size parameters so that only the probe clusters are detected.

The area for each detected particle is displayed in a table.

11. Visually inspect all of the detected particles and exclude the particles that do not look like a real signal.

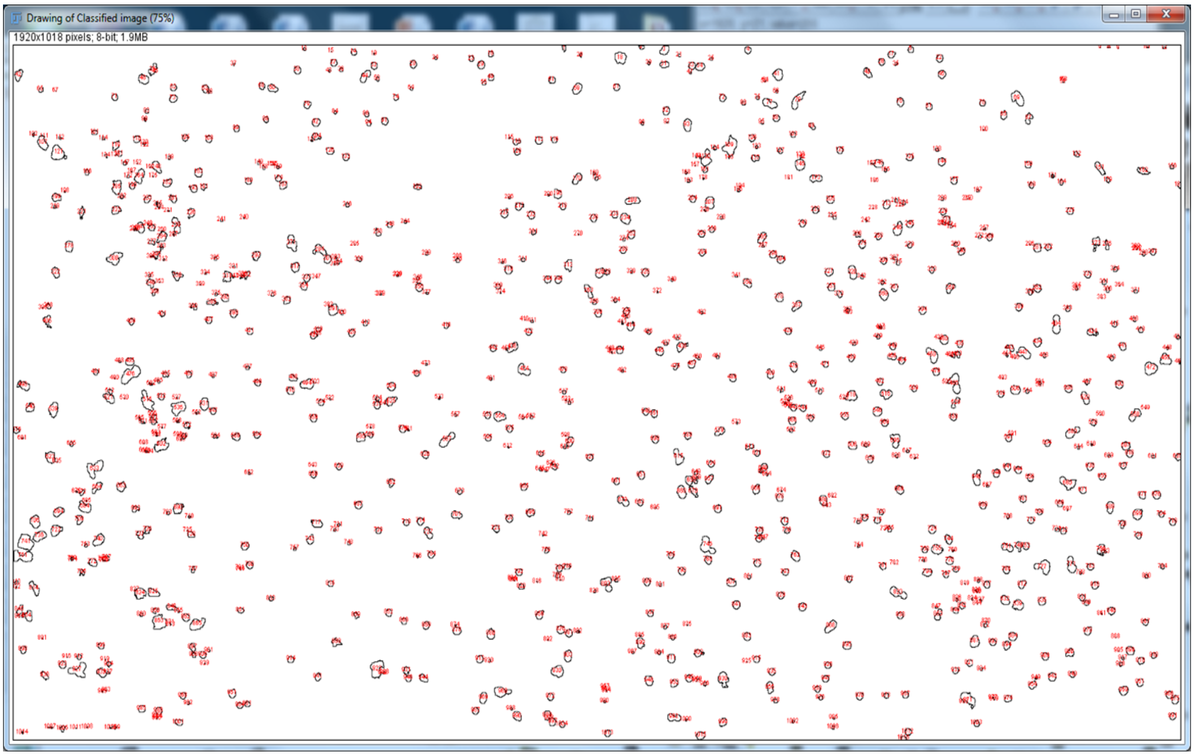

Results

File Edit Font Results

	Area	Mean	Min	Max
2818	85	0	0	0
2819	83	0	0	0
2820	1	0	0	0
2821	1	0	0	0
2822	53	0	0	0
2823	1	0	0	0
2824	7	0	0	0
2825	2	0	0	0
2826	28	0	0	0
2827	28	0	0	0
2828	1	0	0	0
2829	1	0	0	0
2830	8	0	0	0
2831	5	0	0	0
2832	1	0	0	0
2833	1	0	0	0

The total number of signals is 2855

And from A we obtained 585 nuclei

To obtain the total probe count within each probe cluster, determine the area of a single probe using the image and accompanying results table, then divide the area of the larger clusters by the single probe area.

12. Click Next to proceed. Ignore any pop-ups that may appear on the screen.

13. To calculate the number of dots per cell, divide the total number of probe count obtained in Part B by the number of cells obtained in Part A.

2855/585 =4.85 ≈5 therefore the score of this core is 2+

Manual scoring of BaseScope RNA ISH

0 = if no signal or <1/10 tumour cells in high power objective (40X)

1+ = 1-3 dots/cell in 40X objective

2+ = 4-10/cell in 40X objective

3+ = >10/cell with or without <10 cluster dots in 20X

4+= >10/cell with ≥10 cluster dots in 20X
